# Phylogeny, divergence time and historical biogeography of *Hyphoderma* (Hyphodermataceae, Polyporales): Introducing five new species from China

**DOI:** 10.3897/mycokeys.129.183371

**Published:** 2026-02-19

**Authors:** Wen Li, Haijiao Wang, Xiyan Wang, Xiuhe Liao, Kaisheng Wang, Wijesinghe Arachchige Subodini Nuwanthika, Changlin Zhao

**Affiliations:** 1 Key Laboratory of Forest Disaster Warning and Control in Universities of Yunnan Province, Southwest Forestry University, Kunming 650224, China Department Microbial Drugs (MWIS), Helmholtz-Centre for Infection Research Braunschweig Germany; 2 College of Forestry, Southwest Forestry University, Kunming 650224, China College of Forestry, Southwest Forestry University Kunming China; 3 Department Microbial Drugs (MWIS), Helmholtz-Centre for Infection Research, 38124 Braunschweig, Germany Key Laboratory of Forest Disaster Warning and Control in Universities of Yunnan Province, Southwest Forestry University Kunming China; 4 Modern Industry School of Edible-fungi, Southwest Forestry University, Kunming 650224, China Modern Industry School of Edible-fungi, Southwest Forestry University Kunming China

**Keywords:** Biogeographic patterns, molecular systematics, morphology, new taxa, reconstructing ancestral states, wood-decaying fungi

## Abstract

Species of *Hyphoderma* are important wood-inhabiting fungi and play a crucial role in forest ecosystems. Although species diversity within this genus has been increasingly documented in recent years, studies on its origin, evolutionary history, and biogeography remain limited. In this study, we compiled a global dataset of *Hyphoderma* sequences and reconstructed an updated phylogeny, divergence times, and biogeographic history of the genus *Hyphoderma* based on the internal transcribed spacer (ITS) region and nuclear large ribosomal subunit (nLSU) sequences. In addition, integrated morphological and phylogenetic analyses revealed five new *Hyphoderma* species. Molecular clock estimates indicated that the ancestor of *Hyphoderma* likely originated in the Cretaceous, with a mean stem age of 117.76 Mya (95% HPD = 92.38–147.74 Mya). Biogeographic reconstruction further suggested that Asia is the most probable ancestral area of *Hyphoderma* species. This study provides the first comprehensive inference of divergence times, biogeography, and speciation patterns within *Hyphoderma*.

## Introduction

The class Agaricomycetes is a diverse class of fungi within the Basidiomycota, encompassing mushrooms, bracket fungi, puff balls, and other fruiting body-forming species, which are found on all continents, including Antarctica, in habitats ranging from tropical rainforests to arctic and alpine ecosystems, and they have important ecological roles as decayers, mycorrhizal symbionts, and parasites of plants and fungi (Nagy et al. 2025). Saprotrophic Agaricomycetes are broadly categorized into wood decayers and litter decomposers, in which wood-decayer Agaricomycetes are further classified into white rot and brown rot based on the chemical and anatomical characteristics of the decayed wood (Floudas 2021). The genus *Hyphoderma* Wallr. (Hyphodermataceae, Polyporales), typified by *H. setigerum* (Fr.) Donk, represents one of the most speciose and taxonomically challenging groups of wood-decaying fungi (Donk 1957; Nilsson et al. 2003; Kirk et al. 2008; Yurchenko and Wu 2015). Species of *Hyphoderma* typically produce resupinate to effuse-reflexed basidiomata with a ceraceous consistency, smooth to tuberculate or hydnoid hymenophores, a monomitic (rarely dimitic) hyphal system with clamp connections, with or without cystidia, suburniform to subcylindrical basidia, and smooth, thin-walled, ellipsoid to subglobose basidiospores (Bernicchia and Gorjón 2010). Species in this genus are white rot fungi (Wu 1997; Floudas et al. 2012; Guan et al. 2021; Duan et al. 2023; Yang et al. 2023). Currently, 217 specific and infraspecific names are recorded in Index Fungorum (accessed 7 February 2026), of which 125 are accepted worldwide, and 41 species are accepted in China (Nakasone 2008; Wu et al. 2010; Martín et al. 2018; Ma et al. 2021; Yang et al. 2023; Su et al. 2024; Li et al. 2025).

The traditionally circumscribed *Hyphoderma* was shown to be polyphyletic, and 20 species were transferred to *Peniophorella* within the Hymenochaetales (Larsson 2007). Subsequent studies further examined the delimitation of *Hyphoderma* and related genera, revealing that eight *Peniophorella* species form a distinct, well-supported clade separate from *Hyphoderma* (Tellería et al. 2012; Justo et al. 2017). Molecular systematic analyses have since provided a family-level phylogenetic framework for the order Polyporales and demonstrated that Hyphodermataceae contains only a single genus, *Hyphoderma*. A recent identification key to Chinese *Hyphoderma* species was published by Guan et al. (2021). Furthermore, multi-marker phylogenetic analyses based on ITS, nLSU, mtSSU, *rpb*1, and *rpb*2 sequence data revealed that *Hyphoderma* comprises several well-supported clades (Yang et al. 2023, 2025b).

Wood-decay fungi, as a group of macrofungi, possess significant economic and ecological value beyond their taxonomic importance. Biogeographical studies that address the origin, diversification, and distribution patterns of organisms are essential at the genus level for wood-decay fungi (Savinova et al. 2019; He et al. 2024; Hyde et al. 2024; Zhao et al. 2025). Recent advances have substantially improved our understanding of species diversity and divergence times in fungi, including many genera within the Agaricomycetes (He et al. 2019; Varga et al. 2019; Wang et al. 2023; Cui et al. 2025). Currently, more than 10,000 macrofungi species have been described (Hawksworth and Lücking 2017; Niskanen et al. 2023; Hibbett et al. 2025). Elucidating their origins, reconstructing evolutionary histories, and clarifying geographical distributions provide a strong foundation for forest ecosystem research and management (He et al. 2024; Zhao et al. 2025). Although the origin, evolution, and biogeography of certain genera, such as *Porodaedalea* and *Laetiporus*, have been well studied (Song and Cui 2017; Zhao et al. 2025), comparable research remains limited for many other genera, including *Hyphoderma*.

This research continues our long-term investigation into the taxonomy, phylogeny, and fungal diversity of wood-decaying fungi (Dai 2012; Mao et al. 2023; Cho et al. 2023; Pagin-Cláudio and Gugliotta 2024). The study aims to characterize novel and rare fungi, refine phylogenetic relationships within *Hyphoderma*, and provide new insights into the divergence times and biogeographical history of the genus. It is based on an integrative approach combining morphology and molecular phylogenetics. In addition, five new *Hyphoderma* species were discovered in Yunnan Province, China. This research expands the taxonomic framework of *Hyphoderma* through modern taxonomic approaches and enhances our understanding of fungal evolution and geography.

## Materials and methods

### Sample collection and herbarium specimen preparation

Fresh basidiomata of fungi growing on angiosperm branches were collected from Yunnan Province, China. The samples were photographed in situ, metadata were recorded (Rathnayaka et al. 2025), and fresh macroscopic details were documented following the guidelines provided by Dong et al. (2024). All photographs were stacked and merged using Helicon Focus Pro 7.7.5 software. Specimens were dried in an electric food dehydrator at 40 °C, then sealed and stored in envelope bags (Dong et al. 2024, 2025), and deposited in the herbarium of Southwest Forestry University (**SWFC**), Kunming, Yunnan Province, P.R. China.

### Morphological examinations

Macro-morphological descriptions were based on field notes and photographs captured in the field and laboratory. The descriptions followed Petersen (1996) for color terminology. Micro-morphological characters were obtained from dried specimens observed under a light microscope at 1000× oil immersion (Dong et al. 2024, 2025). Sections were mounted in 5% KOH, 1% Congo Red solution, and 1% phloxine B (C_20_H_2_Br_4_Cl_4_Na_2_O_5_). Other reagents, including Cotton Blue and Melzer’s reagent, were also used to observe micromorphology following the methods of Wu et al. (2019, 2022). To show variation in spore sizes, 5% of measurements were excluded from each end of the range and are shown in parentheses. At least 30 basidiospores from each specimen were measured. Stalks were excluded from basidia measurements, and the hilar appendage was excluded from basidiospore measurements (Yuan and Zhao 2024). The following abbreviations were used: **KOH** = 5% potassium hydroxide aqueous solution; **CB** = Cotton Blue; **CB–** = acyanophilous; **CB+** = cyanophilous; **IKI** = Melzer’s reagent; **IKI–** = both inamyloid and indextrinoid; **L** = mean spore length (arithmetic average of all spores); **W** = mean spore width (arithmetic average of all spores); **Q** = variation in the L/W ratios between the specimens studied; and **n** = a/b (number of spores (a) measured from a given number (b) of specimens).

### DNA extraction, PCR amplification, sequencing

The CTAB rapid fungal genome extraction kit DN14 (Aidlab Biotechnologies Co., Ltd., Beijing, China) was used to obtain genomic DNA from dried fungal specimens according to the manufacturer’s instructions. The extracted DNA was maintained at −20 °C for long-term storage. Two molecular markers were investigated: the internal transcribed spacer (ITS) region and the nuclear large subunit ribosomal RNA (LSU) gene. PCR primers and conditions are shown in Table 1. PCR products were purified and sequenced at Kunming Tsingke Biological Technology Limited Company (Yunnan Province, China). All newly generated sequences were deposited in NCBI GenBank (Sayers et al. 2025) (Table 1).

**Table 1.** Taxa information and GenBank accession numbers used in this study [* indicates type materials; — indicates sequence unavailability].

Species Name	Sample No.	GenBank Accession No.		References	Country
		ITS	nLSU		
*Amylocorticium cebennense*	HHB-2808	GU187505	GU187561	Wang et al. 2023	USA
*Anomoloma myceliosum*	MJL-4413	GU187500	GU187559	Wang et al. 2023	USA
*Aroramyces gelatinosporus*	H4010	–	DQ218524	Wang et al. 2023	USA
*Athelia arachnoidea*	CBS 418.72	GU187504	GU187557	Wang et al. 2023	Netherlands
*Auricularia heimuer*	Xiaoheimao	LT716074	KY418890	Wang et al. 2023	China
*Boletopsis leucomelaena*	AFTOL-ID 1527	DQ484064	DQ154112	Wang et al. 2023	USA
*Calocera cornea*	AFTOL 438	AY789083	AY701526	Wang et al. 2023	USA
*Coltricia abieticola*	Cui 10321	KX364785	KX364804	Wang et al. 2023	China
*Coniferiporia qilianensis*	Dai 13320	MT416471	MT386051	Wang et al. 2023	China
*Coniophora arida*	FP104367	GU187510	GU187573	Wang et al. 2023	USA
*Corticium boreoroseum*	MG 42	MW805842	MW805816	Wang et al. 2023	Sweden
*Dacryopinax spathularia*	AFTOL 454	AY854070	AY701525	Wang et al. 2023	USA
*Diplomitoporus crustulinus*	FD-137	KP135299	KP135211	Justo et al. 2017	USA
*Echinodontium tinctorium*	AFTOL-ID 455	AY854088	AF393056	Wang et al. 2023	USA
*Fomitopsis ostreiformi*	Cui 18217	OL621855	OL621244	Wang et al. 2023	China
*F. subtropica*	Dai 18566	OL621854	OL621243	Wang et al. 2023	China
*Gomphidius roseus*	MB 95-038	DQ534570	DQ534669	Wang et al. 2023	Germany
*Heterobasidion annosum*	06129/6	KJ583211	KJ583225	Wang et al. 2023	China
Hyphoderma alboarachnum*	**CLZhao 30488**	**PV470563**	**—**	**Present study**	**China**
*H. amoenum*	USO 286622	HE577030	—	Tellería et al. 2012	Canada
H. asianum*	CLZhao 18091	OR141726	PP826262	Yang et al. 2025b	China
*H. assimile*	CBS:125852	MH863808	MH875272	Vu et al. 2019	New Zealand
H. australosetigerum*	MA-Fungi 92235	MN963764		Boonmee et al. 2021	Chile
*H. australosetigerum*	MA-Fungi 92240	MN963760		Boonmee et al. 2021	Chile
H. bambusinum*	**CLZhao 29903**	**PV469674**	**PV819428**	**Present study**	**China**
H. cinereofuscum*	CLZhao 30341	PQ492371	PQ511128	Li et al. 2025	China
*H. cinereofuscum*	CLZhao 30283	PQ492372	PQ511129	Li et al. 2025	China
*H. cremeoalbum*	NH 11538 (GB)	DQ677492	DQ677492	Larsson 2007	Sweden
*H. cremeoalbum*	CLZhao 17007	OM985716	OM985753	Duan et al. 2023	China
H. crystallinum*	CLZhao 9338	MW917161	MW913414	Guan and Zhao 2021a	China
*H. crystallinum*	CLZhao 9374	MW917162	MW913415	Guan and Zhao 2021a	China
*H. definitum*	NH 12266 (GB)	DQ677493	DQ677493	Larsson 2007	China
*H. fissuratum*	CLZhao 6731	MT791331	—	Ma et al. 2021	China
H. fissuratum*	CLZhao 6726	MT791330	MT791334	Ma et al. 2021	China
H. floccosum*	CLZhao 17129	MW301683	MW293733	Guan and Zhao 2021b	China
*H. floccosum*	CLZhao 17215	MW301687	MW293735	Guan and Zhao 2021b	China
***H. fulgens***	**CLZhao 30254**	**PV469670**	**PV819427**	**Present study**	**China**
***H. fulgens***	**CLZhao 36600**	**PV829546**	**PV810096**	**Present study**	**China**
H. fulgens*	**CLZhao 37429**	**PV829544**	**PV810095**	**Present study**	**China**
***H. fulgens***	**CLZhao 39474**	**PV829547**	**PV810098**	**Present study**	**China**
*H. granuliferum*	5273	JN710545	JN710545	Yurchenko and Wu 2014a	Canada
H. grandineum*	**CLZhao 30046**	**PV470561**	**PV819429**	**Present study**	**China**
***H. grandineum***	**CLZhao 43328**	**PV470562**	**PV819430**	**Present study**	**China**
H. guangdongense*	CLZhao 12657	PP235513	PP235514	Su et al. 2024	China
*H. incrustatum*	KHL6685	—	AY586668	Yurchenko and Wu 2014a	Spain
***H. laceratum***	**CLZhao 34242**	**PV829552**	**PV810101**	**Present study**	**China**
***H. laceratum***	**CLZhao 34672**	**PV829553**	**PV810103**	**Present study**	**China**
***H. laceratum***	**CLZhao 34958**	**PV829556**	**PV810106**	**Present study**	**China**
***H. laceratum***	**CLZhao 34961**	**PV829557**		**Present study**	**China**
*H. litschaueri*	NH 7603 (GB)	DQ677496	DQ677496	Larsson 2007	Sweden
*H. litschaueri*	FP-101740-Sp	KP135295	KP135219	Floudas and Hibbett 2015	USA
*H. macaronesicum*	MA:Fungi 90388	KC984327	—	Tellería et al. 2012	USA
*H. macaronesicum*	TFC:Mic 15115	HE577011	—	Yurchenko and Wu 2014b	China
H. marginatum*	CLZhao 3404	OM985717	OM985754	Duan et al. 2023	China
*H. medioburiense*	FD-335	KP135298	KP135220	Floudas and Hibbett 2015	China
H. membranaceum*	CLZhao 5844	MW917167	MW913420	Guan and Zhao 2021a	China
*H. membranaceum*	CLZhao 6971	MW917168	MW913421	Guan and Zhao 2021a	China
*H. microporoides*	CLZhao 6857	MW917169	MW913422	Guan and Zhao 2021a	China
*H. microporoides*	CLZhao 8695	MW917170	MW913423	Guan and Zhao 2021a	China
H. moniliforme*	Wu 0211-42	KC928282	—	Yurchenko and Wu 2015	China
*H. moniliforme*	Wu 0211-46	KC928284	—	Yurchenko and Wu 2015	China
H. mopanshanense*	CLZhao 6498	MT791329	MT791333	Ma et al. 2021	China
*H. mopanshanense*	CLZhao 6449	OM985720	OM985759	Duan et al. 2023	China
*H. nemorale*	TNM F3931	KJ885183	KJ885184	Yurchenko and Wu 2015	China
*H. nemorale*	Wu 9508-14	KC928280	KC928281	Yurchenko and Wu 2015	China
H. niveomarginatum*	CLZhao 25078	OR141728	OR506179	Yang et al. 2023	China
*H. nudicephalum*	Wu9307_29	AJ534269	—	Nilsson et al. 2003	China
*H. nudicephalum*	CLZhao 17839	OM985721	OM985760	Duan et al. 2023	China
*H. obtusiforme*	KHL1464	JN572909	—	Yurchenko and Wu 2014b	Spain
*H. obtusiforme*	KHL11105	JN572910	—	Yurchenko and Wu 2014b	Spain
*H. obtusum*	JS17804	—	AY586670	Yurchenko and Wu 2014b	China
*H. occidentale*	KHL 8477 (GB)	DQ677499	DQ677499	Larsson 2007	China
H. paramacaronesicum*	MA:Fungi 87736	KC984399	—	Martín et al. 2018	USA
*H. paramacaronesicum*	MA:Fungi 87737	KC984405	—	Martín et al. 2018	China
*H. pinicola*	Wu 0108-32	KJ885181	KJ885182	Yurchenko and Wu 2014b	China
*H. pinicola*	Wu 0108-36	KC928278	KC928279	Yurchenko and Wu 2014b	China
*H. prosopidis*	ARIZ HHB 8479	HE577029	—	Yurchenko and Wu 2015	China
H. puerense*	CLZhao 9476	MW443045	—	Guan et al. 2021	China
*H. puerense*	CLZhao 9583	MW443046	MW443051	Guan et al. 2021	China
H. qujingense*	CLZhao 26018	OR141729	PP826263	Yang et al. 2025b	China
*H. roseocremeum*	NH 10545		AY586672	Yurchenko and Wu 2014a	Sweden
*H. setigerum*	FCUG 1200	AJ534273	—	Nilsson et al. 2003	Sweden
*H. setigerum*	FCUG 1688	AJ534272	—	Nilsson et al. 2003	Sweden
H. sinense*	CLZhao 7963	MW301679	MW293730	Guan and Zhao 2021b	China
*H. sinense*	CLZhao 17811	MW301682	MW293732	Guan and Zhao 2021b	China
*H. sordidum*	CLZhao 27379	OR141731	—	Yang et al. 2023	China
H. sordidum*	CLZhao 27390	OR141732	OR506180	Yang et al. 2023	China
*H. subsetigerum*	HHB11620	GQ409521	—	Yurchenko and Wu 2014a	China
H. tenuissimum*	CLZhao 7221	MW443049	MW443054	Guan et al. 2021	China
*H. tenuissimum*	CLZhao 16210	MW443050	MW443055	Guan et al. 2021	China
*H. transiens*	NH 12304	DQ677504	DQ677504	Larsson 2007	Sweden
H. tropicum*	CLZhao 17308	OM985727	OM985768	Duan et al. 2023	China
*H. variolosum*	CBS:734.91	MH862320	MH873992	Vu et al. 2019	China
*H. variolosum*	CBS:735.91	MH862321	MH873993	Vu et al. 2019	China
H. weishanense*	CLZhao 22403	OR141727	OR506181	Yang et al. 2023	China
*H. yunnanense*	CLZhao 8845	OM985728	OM985769	Duan et al. 2023	China
*Hyphodontia pachyspora*	LWZ 20170908-5	MT319426	MT319160	Wang et al. 2023	China
*Hymenochaete sphaericola*	LWZ20190808-2b	ON063656	ON063855	Wang et al. 2023	China
*Jaapia argillacea*	CBS 252.74	GU187524	GU187581	Wang et al. 2023	Netherlands
*Laetisaria fuciformis*	CBS:182.49	MH856485	MH868023	Vu et al. 2019	Netherlands
*Leptosporomyces raunkiaerii*	HHB-7628	GU187528	GU187588	Wang et al. 2023	USA
*Lyomyces incanus*	CLZhao 22900	OR094481	OR449936	Dong et al. 2024	China
*Meripilus giganteus*	FP 135344	KP135307	KP135228	Wang et al. 2023	USA
*Neurospora crassa*	VB2	HQ271348	AF286411	Wang et al. 2023	India
*Nigrofomes sinomelanoporus*	Cui 5277	MF629836	MF629832	Wang et al. 2023	China
*Phanerochaete aculeata*	Wu 880701-2	MZ422787	GQ470636	Wang et al. 2023	China
*Phanerochaete mopanshanensis*	CLZhao 2357 *	OR096190	OR461450	Dong et al. 2024	China
*Physisporinus longicystidius*	Cui 16630	ON417177	ON417227	Wang et al. 2023	China
*Polyporus squamosus*	Cui 10595	KU189778	KU189809	Wang et al. 2023	China
*Polyporus varius*	Cui 12249	KU507581	KU507583	Wang et al. 2023	China
*Peniophorella subpraetermissa*	LWZ 20190816-3b	ON063689	ON063889	Wang et al. 2023	China
*Rigidoporus populinus*	LWZ 20190811-39a	ON063674	ON063874	Wang et al. 2023	China
*Steccherinum weishanense*	CLZhao 24911 *	OR096207	OR461456	Dong et al. 2024	China
*S. ochraceum*	KHL 11902	JN710590	JN710590	Wang et al. 2023	Sweden
*Thelephora ganbajun*	ZRL20151295	LT716082	KY418908	Wang et al. 2023	China
*Xylodon olivaceobubalinus*	CLZhao 25174	OR167772	OR449948	Dong et al. 2024	China

### Phylogenetic analyses

ITS and nLSU sequences were aligned using MAFFT version 7 (Katoh et al. 2019) with the G-INS-i strategy. Single-gene alignments were manually adjusted using AliView version 1.27 (Larsson 2014; Caboň et al. 2019). The ITS and nLSU sequences were then combined using Mesquite version 3.51. The final sequence alignment, together with related information, was deposited in Figshare (doi: https://doi.org/10.6084/m9.figshare.30939239). *Diplomitoporus crustulinus* (Bres.) Domanski was selected as the outgroup for phylogenetic analyses following Justo et al. (2017). Maximum parsimony (MP), maximum likelihood (ML), and Bayesian inference (BI) analyses were performed using the combined dataset. Phylogenetic analytical procedures followed Sun et al. (2022). MP analysis was conducted in PAUP* version 4.0b10 (Swofford 2002). All characters were equally weighted, and gaps were treated as missing data. Trees were inferred using a heuristic search with TBR branch swapping and 1,000 random sequence additions. Maxtrees were set to 5,000, branches of zero length were collapsed, and all most-parsimonious trees were saved.

Clade robustness was assessed using bootstrap (BT) analysis with 1,000 replicates (Felsenstein 1985; Guan et al. 2023). Descriptive tree statistics, including tree length (TL), consistency index (CI), retention index (RI), rescaled consistency index (RC), and homoplasy index (HI), were calculated for each most-parsimonious tree. ML analysis was conducted using RAxML-HPC2 through the CIPRES Science Gateway (www.phylo.org) (Miller et al. 2012). Branch support (BS) for ML analysis was estimated using 1,000 bootstrap replicates under the gamma model.

**Figure 1.**
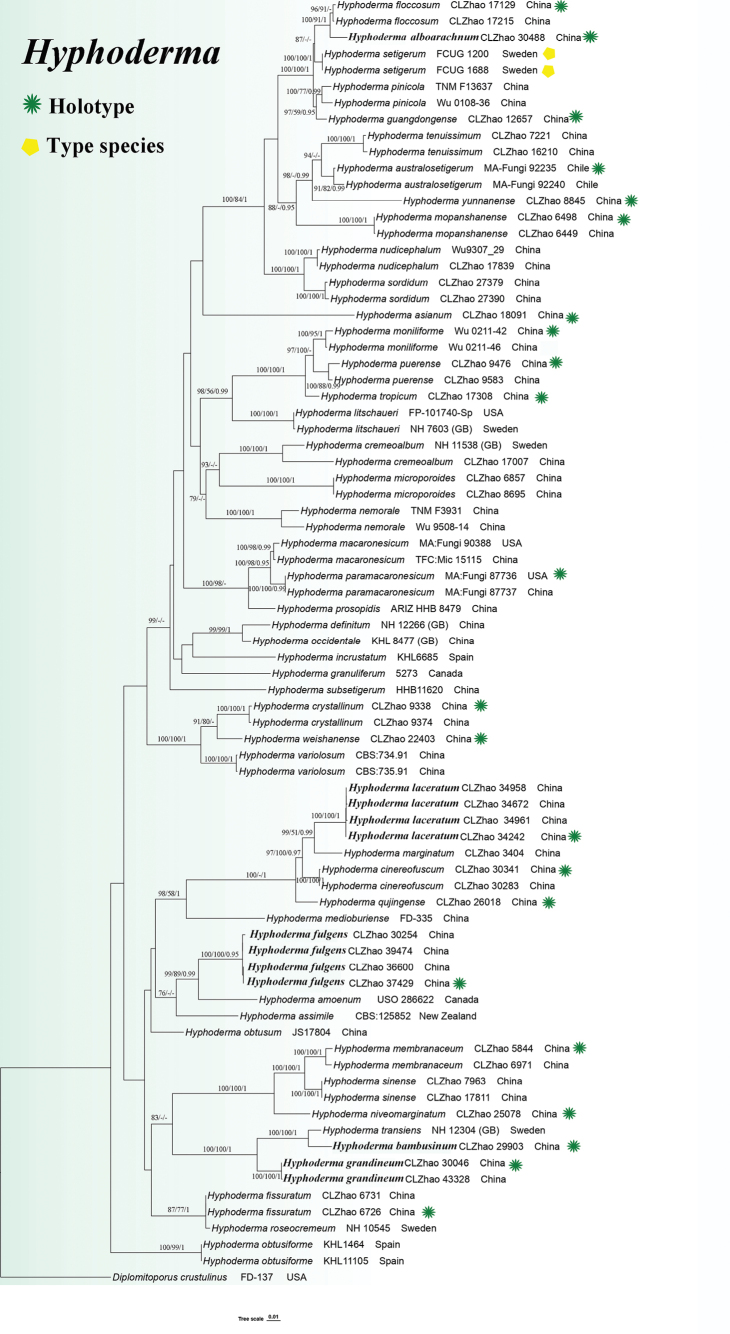
Phylogenetic tree generated from an ML analysis based on ITS+nLSU sequences. Branches are labeled with maximum likelihood bootstrap values equal to or higher than 70%, maximum parsimony bootstrap values equal to or higher than 50%, and Bayesian posterior probabilities equal to or higher than 0.95. Novel sequences are printed in bold.

MrModeltest 2.3 (Nylander 2004) was used to determine the best-fit evolutionary model for each dataset. BI was performed in MrBayes 3.2.7a using the GTR+I+G model of DNA substitution with gamma-distributed rate variation across sites (Ronquist et al. 2012). Four Markov chains were run for two independent runs from random starting trees for four million generations for the ITS+nLSU dataset (Fig. 1), with trees and parameters sampled every 1,000 generations. The first one-fourth of all generations was discarded as burn-in. A majority-rule consensus tree was calculated from the remaining trees. Branches were considered significantly supported if they received a maximum likelihood bootstrap value (BS) ≥ 70%, a maximum parsimony bootstrap value (BT) ≥ 50%, or a Bayesian posterior probability (BPP) ≥ 0.95.

### Estimation of divergence times

The divergence-time analysis followed methodologies outlined in previous studies (Wang et al. 2023; Cui et al. 2025). Using a secondary calibration approach, two independent analyses were conducted to estimate divergence times within *Hyphoderma*.

BEAST v2.6.0 was used to perform molecular clock analyses on the combined alignment derived from Table 1 (Sun et al. 2022; Wang et al. 2023; Cui et al. 2025). A lognormal relaxed molecular clock model and a Yule speciation prior were selected to estimate divergence times and their associated credibility intervals (Zhao and Wu 2017; Spirin et al. 2024). Two calibration points were applied. The first calibration used an offset age with a gamma distribution prior (scale = 20, shape = 1) for Basidiomycota at 400 Mya, based on the divergence between Ascomycota and Basidiomycota inferred from the fossil *Paleopyrenomycites devonicus*, discovered in Great Britain (Floudas et al. 2012). Trees were sampled every 1,000 generations over 200 million generations, with the first 10% discarded as burn-in. The resulting log files were examined for convergence using Tracer v1.5 (Wang et al. 2023).

Similarly, divergence times of species within *Hyphoderma* were estimated based on the results obtained above. This analysis included 48 samples representing a single genus of *Hyphoderma*, with *Meripilus giganteus* and *Physisporinus longicystidius* designated as outgroup taxa. Sequence data used in this analysis are provided in Table 2. These analyses were used to estimate the origin and biogeographical history of *Hyphoderma* species.

**Table 2.** Morphological comparison of new species with similar taxa of the genus *Hyphoderma*.

Species name	Basidiomata	Hyphal system	Cystidia	Basidiospores	References
*Hyphoderma alboarachnum*	Membranaceous, hymenial surface arachnoid.	Monomitic; generative hyphae with clamp connections, colorless, thin-walled.	Absent	Oblong-ellipsoid to cylindrical; 5–6 × 2–3 µm.	Present study
*H. asianum*	Ceraceous to membranaceous, hymenial surface smooth, floccose.	Monomitic; generative hyphae with clamp connections, colorless, thin-walled.	Moniliform; 20–50 × 4–6 μm.	Ellipsoid; 6.5–10 × 4–6 μm.	Yang et al. 2025b
*H. bambusinum*	Membranaceous, hymenial surface smooth.	Monomitic; generative hyphae with clamp connections, colorless, thin–to thick.	Tubular; 21.5–26.5 × 5–6.5 µm.	Ellipsoid; 4.5–6.5 × 3–4.5 µm.	Present study
*H. cinereofuscum*	Coriaceous, hymenial surface smooth.	Monomitic; generative hyphae with simple-septa, colorless, thin-walled.	1) Tubular; 25.9–37.1 × 1.8–2.5 μm 2) Capitate; 12.5–15.5 × 6.2–7 μm.	Cylindrical; 9–11.5 × 4.5–5 μm.	Li et al. 2025
*H. crystallinum*	Membranaceous, scattered nubby crystals.	Monomitic; generative hyphae with clamp connections, colorless, thin-walled.	1) Tubular 32–51 × 5–10 μm. 2) Encrusted 14–46 × 4–11 μm.	Allantoid; 11–14.5 × 4–5.5 μm.	Guan and Zhao 2021a
*H. fissuratum*	Ceraceous, hymenial surface smooth.	monomitic, generative hyphae with clamp connections.	Absent	Cylindrical; 8.5–10.3 × 3–4 μm.	Ma et al. 2021
*H. floccosum*	Ceraceous, hymenial surface farinaceous.	Monomitic; generative hyphae with simple-septa, colorless, thin-walled.	1) septate 60–161 × 5.5–10 μm. 2) tubular 37.5–100 × 4–8.5 μm.	Ellipsoid to allantoid; 6–9.5 × 3–4.5 μm.	Guan and Zhao 2021b
*H. fulgens*	Membranaceous, hymenial surface smooth.	Monomitic; generative hyphae with simple-septa, colorless, thin-walled.	Clavate 37.1 × 5.5–7.5 µm.	Subcylindrical; 7.5–11.5 × 2.5–4 µm.	Present study
*H. grandineum*	Membranaceous, hymenial surface grandinioid.	Monomitic; generative hyphae with simple-septa, colorless, thin-walled.	Leptocystidia 29.5–33.5 × 4–5 µm.	Cylindrical;7–9 × 2.5–4 µm.	Present study
*H. laceratum*	Membranaceous, hymenial surface smooth.	Monomitic; generative hyphae with simple-septa, colorless, thin-walled.	1) Clavate 27–29 × 6.5–8 µm. 2) Capitate 26–30 × 7.5–8.5 µm.	Subellipsoid; 6.5–9 × 3–4.5 µm.	Present study
*H. marginatum*	Membranaceous, hymenial surface smooth.	Monomitic; generative hyphae with simple-septa, colorless, thin-walled.	Cylindrical 30–48.5 × 7.5–11.5 µm.	Cylindrical; 9–10 × 3.5–4.5 µm.	Duan et al. 2023
*H. membranaceum*	Membranaceous, hymenial surface tuberculate	Monomitic, generative hyphae with clamp thin-walled.	Moniliform; 28–60 × 6.5–10.5 μm.	Ellipsoid to cylindrical 11–13.5 × 4.5–5.5 μm.	Guan and Zhao 2021a
*H. microporoides*	Cottony hymenial surface smooth.	Monomitic, generative hyphae with clamp connections, colorless, thin-walled	Capitate; 18–51 × 4.5–7 μm.	Cylindrical to allantoid, 8.5–10 × 2.5–3.5 μm.	Guan and Zhao 2021a
*H. mopanshanense*	Ceraceous, hymenial surface porulose to pilose.	monomitic, generative hyphae bearing clamp connections	Fusiform; 86–171 × 10.5–13 μm	Cylindrical 7.8–9.7 × 2.6–3.3 μm.	Ma et al. 2021
*H. niveomarginatum*	Ceraceous, hymenial surface smooth.	Monomitic, generative hyphae with clamp connections, colorless, thin-walled	Moniliform; 29–55.5 × 5–7 µm.	Ellipsoid; 7–9 × 3.5–5 μm.	Yang et al. 2023
*H. pinicola*	Membranaceous, hymenial surface chalky.	Monomitic, generative hyphae with clamp connections, colorless, thin-walled	(1) Scattered; 65–180 × 7–11 μm. (2) Aseptate; 35–45 × 6–7 μm.	Cylindrical to allantoid; 13–16 × 4–4.5 μm.	Yurchenko and Wu 2014a
*H. puerense*	Byssoid, hymenial surface smooth, floccose.	Monomitic; generative hyphae with clamp connections, colorless, thick-walled.	Tubular; 25–97 × 5.5–9.5 µm.	Ellipsoid; 6–7.5 × 3–4.5 μm.	Guan et al. 2021
*H. qujingense*	Membranaceous, hymenial surface smooth, floccose.	Monomitic; generative hyphae with clamp connections, colorless, thin-walled.	Capitate; 20–40 × 9–13 μm.	Ellipsoid to cylindrical; 7–11.5 × 3.5–5 μm.	Yang et al. 2025b
*H. sinense*	Membranaceous, hymenial surface smooth.	Monomitic; generative hyphae with clamp connections, colorless, thick-walled.	1) Encrusted; 18.5–38 × 6–11 μm. 2) Moniliform; 30–60.5 × 3–5 μm.	Cylindrical to slightly allantoid; 8–11.5 × 2.6–3.3 μm	Guan and Zhao 2021b
*H. sordidum*	Membranaceous, hymenial surface smooth.	Monomitic; generative hyphae with clamp connections, colorless, thin-walled.	Tubular; 42–72.5 × 6–11 μm.	Ellipsoid; 3–4.5 × 2–3 μm.	Yang et al. 2023
*H. tenuissimum*	Membranaceous, hymenial surface tuberculate to minutely-grandinioid	Monomitic; generative hyphae with clamp connections, colorless, thick-walled.	Cylindrical; 50–220 × 6.5–13 µm.	Cylindrical; 7–10.5 × 3–4.5 µm.	Guan et al. 2021
*H. tropicum*	Coriaceous hymenial surface tuberculate.	Monomitic; generative hyphae with clamp connections, colorless, thin-walled.	Moniliform; 60–102.5 × 5.5–7.5 μm.	Ellipsoid to cylindrical; 6.5–7.5 × 3–4 μm.	Duan et al. 2023
*H. weishanense*	Membranaceous, hymenial surface smooth.	Monomitic; generative hyphae with clamp connections, colorless, thin-walled.	Absent	Broadly ellipsoid; 4.5–8.5 × 4–7 μm.	Yang et al. 2023
*H. yunnanense*	Corneus, hymenial surface tuberculate.	Monomitic; generative hyphae with clamp connections, colorless, thin-walled.	Cylindrical; 63–124 × 7–10 µm	Ellipsoid to cylindrical; 10–11.5 × 4–5.5 μm.	Duan et al. 2023

Divergence-time analyses were further conducted using BEAST v1.10.4 (He et al. 2024). XML input files were generated in BEAUti v1.10.4 by importing separate NEXUS files for each gene partition. Gene partitions were unlinked for substitution and molecular-clock models but linked for gene trees. Nucleotide substitution models were selected using jModelTest v2, with GTR+I+G applied to ITS and nrLSU. An uncorrelated lognormal relaxed clock model and a Yule speciation prior were used to assume a constant speciation rate per lineage. The prior distribution for the ucld.mean parameter was gamma-distributed (shape = 1.0, scale = 0.001, offset = 0.0) for all genes. Secondary calibrations were implemented using a normally distributed prior on the treeModel.rootHeight parameter (SD = 1), with mean values determined by fossil-based node calibrations. Four independent Markov chain Monte Carlo (MCMC) runs were performed, each consisting of 100,000,000 generations, with parameters sampled every 10,000 generations. Log files were assessed for convergence and mixing using Tracer v1.7.1 (Wang et al. 2023) (http://tree.bio.ed.ac.uk/software/tracer/) to confirm effective sample size (ESS) values exceeded 200. An ultrametric maximum clade credibility (MCC) tree, with mean node ages and 95% highest posterior density (HPD) intervals and per-clade posterior probabilities, was summarized using TreeAnnotator v1.10.4, applying a 25% burn-in and a posterior probability threshold of 0.8. Resulting trees were visualized using iTOL v5 (Wang et al. 2023; Zhao et al. 2023; Cui et al. 2025).

### Biogeographic analysis of *Hyphoderma*

Biogeographic analysis of the genus *Hyphoderma* was conducted using Reconstruct Ancestral State in Phylogenies (RASP) v4.2 with the Dispersal–Extinction–Cladogenesis (DEC) model, estimated through the BioGeoBEARS package (Cui et al. 2025). Ancestral area reconstruction was performed using a posterior distribution of the aligned dataset 4, which was estimated in BEAST v2.6.5 with 10 million generations. The geographical distribution of *Hyphoderma* was divided into six regions: A) Asia, B) Europe, C) North America, D) South America, E) Africa, and F) Oceania.

## Results

### Phylogeny of *Hyphoderma*

The ITS+nLSU dataset comprised sequences from 79 fungal specimens representing 48 species. The aligned dataset contained 2,040 characters, of which 1,414 were constant, 107 were variable but parsimony-uninformative, and 519 (25%) were parsimony-informative. Maximum parsimony analysis yielded 4,898 equally parsimonious trees (TL = 3.063, CI = 0.3389, HI = 0.6611, RI = 0.7269, and RC = 0.2463). Bayesian inference and ML analyses produced topologies largely congruent with the MP results, with an average standard deviation of split frequencies of 0.019743 for the BI analysis.

The phylogenetic tree inferred from ITS+nLSU sequences (Fig. 1) revealed that the five new taxa were grouped within the genus *Hyphoderma*. *Hyphoderma alboarachnum* was closely related to *H. floccosum* C.L. Zhao and Q.X. Guan. *Hyphoderma bambusinum* was closely related to *H. transiens* (Bres.) Parmasto. The species *H. fulgens* was closely related to *H. amoenum* (Burt) Donk. *Hyphoderma grandineum* was closely related to both *H. transiens* and *H. bambusinum*. *Hyphoderma laceratum* was closely related to *H. marginatum* Z.Y. Duan and C.L. Zhao.

This study conducted a comparative analysis of the macro- and micromorphological characteristics of the five new species and compared the morphological features with those of other *Hyphoderma* species from Yunnan Province, China (Fig. 7; Table 2).

**Figure 2.**
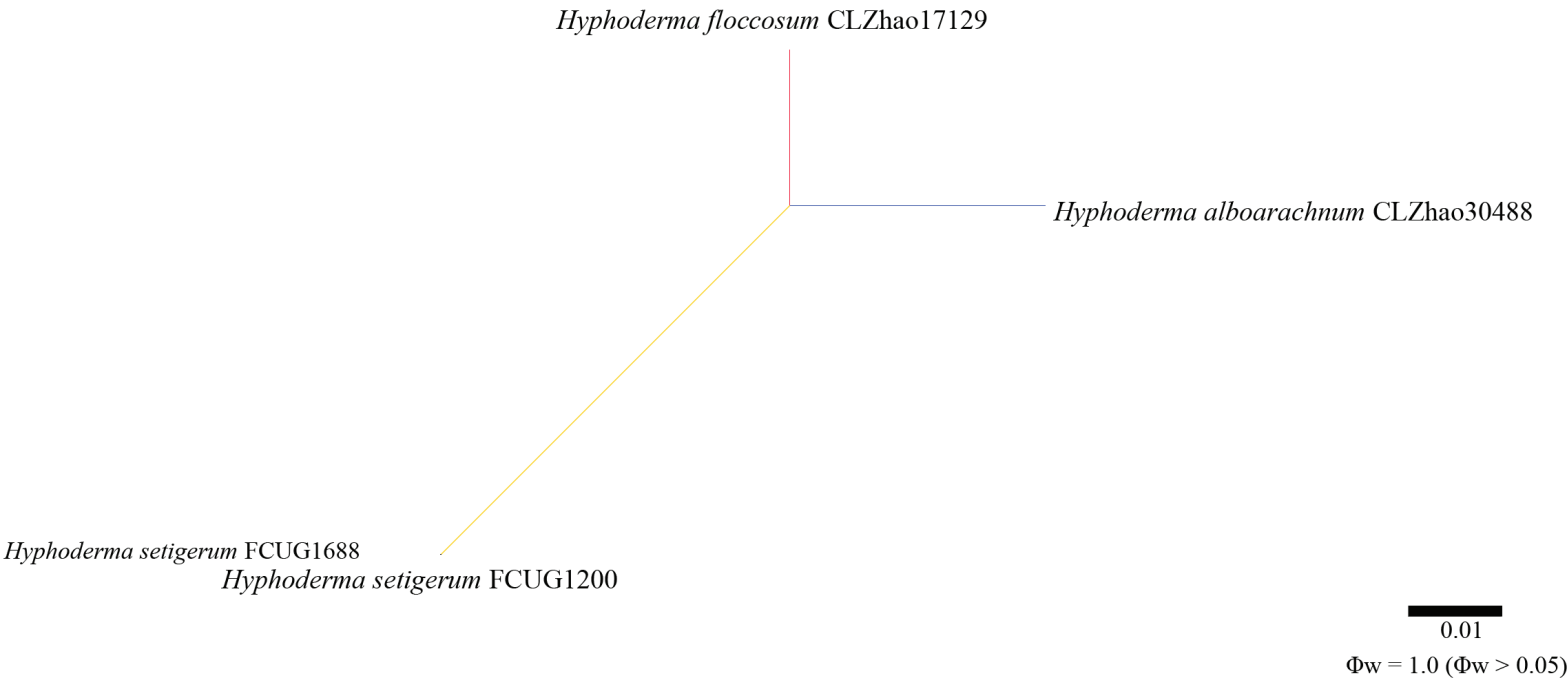
Results of the pairwise homoplasy index (PHI) test for the combined partial ITS sequence data of *Hyphoderma alboarachnum* and closely related taxa, using the LogDet transformation and splits decomposition. PHI test results (Φw ≤ 0.05) indicate significant recombination within the dataset.

**Figure 3.**
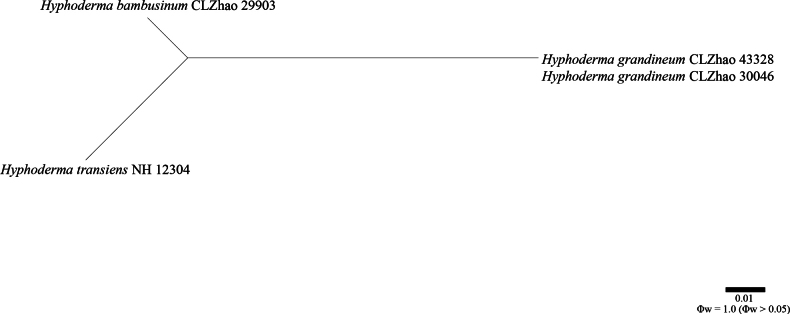
Results of the pairwise homoplasy index (PHI) test for the combined partial ITS sequence data of *Hyphoderma bambusinum* and closely related taxa, using the LogDet transformation and splits decomposition. PHI test results (Φw ≤ 0.05) indicate significant recombination within the dataset.

**Figure 4.**
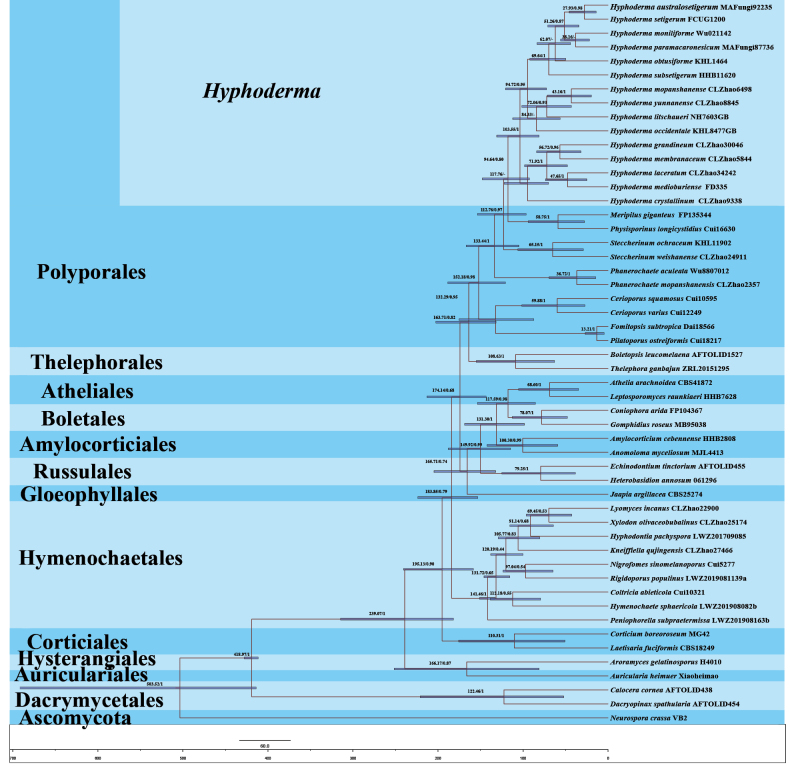
Maximum-clade-credibility chronogram and estimated divergence times of families within *Hyphoderma* inferred from the combined dataset of ITS and nLSU regions. The estimated divergence times of 95% highest posterior density are indicated as node bars for all clades and are also provided in the upper left of the tree as exact numbers for families within Hymenochaetales. Bayesian posterior probabilities above 0.7 and mean divergence times of clades (crown ages) are labeled before and after the slashes, respectively, at the nodes.

**Figure 5.**
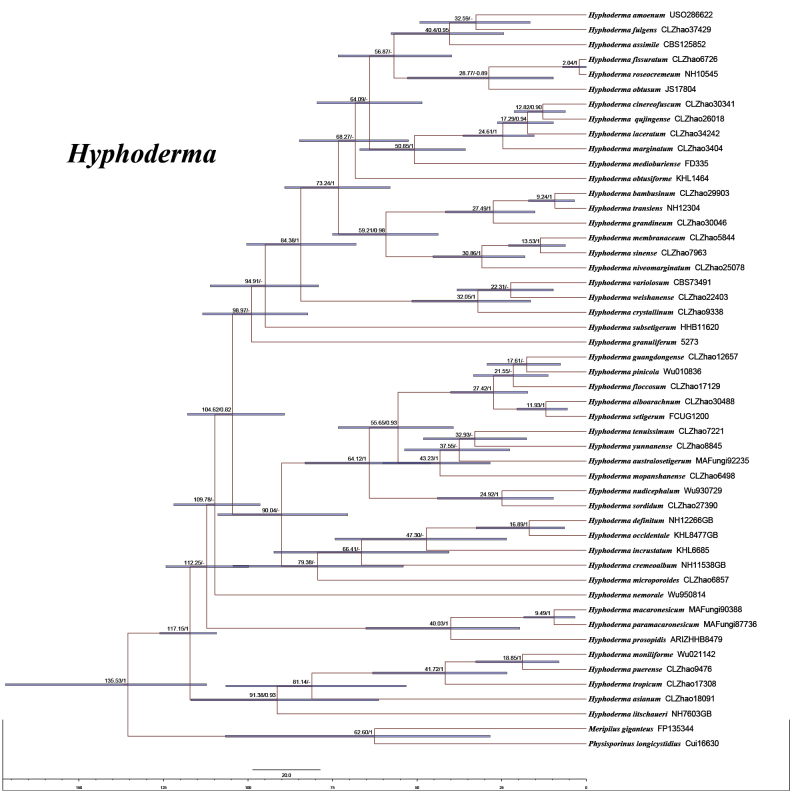
Estimated divergence times for the genus *Hyphoderma*, derived from molecular clock analyses using a combined dataset of internal transcribed spacers (ITS) and nuclear large ribosomal subunit (nLSU) sequences. Mean divergence times (Ma) and posterior probabilities (PP) > 0.8 are annotated at the internodes, with horizontal blue bars representing the 95% highest posterior density (HPD) intervals for divergence time estimates.

**Figure 6.**
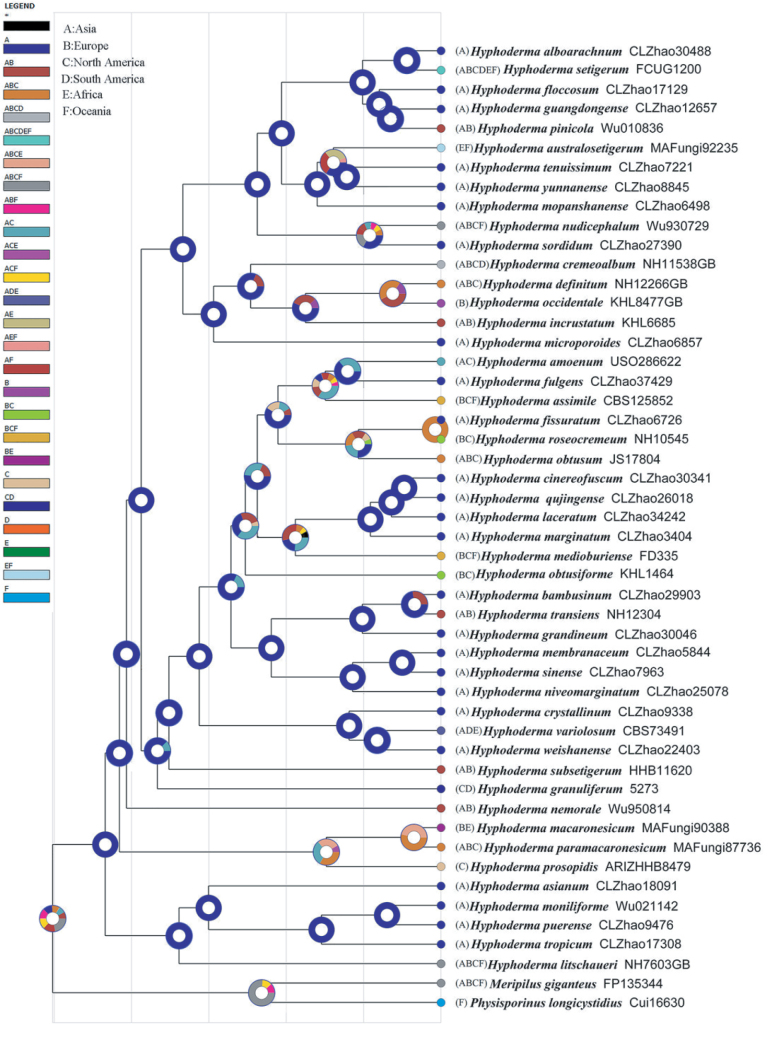
Ancestral state reconstruction of *Hyphoderma* conducted to determine (**A**) the origin center and (**B**) the origin host trees using a dataset comprising internal transcribed spacers (ITS) and nuclear large ribosomal subunit (nLSU) sequences. At each node, a pie chart represents the possible ancestral distributions inferred from dispersal–extinction–cladogenesis (DEC) analyses, as implemented in Reconstruct Ancestral State in Phylogenies (RASP).

**Figure 7.**
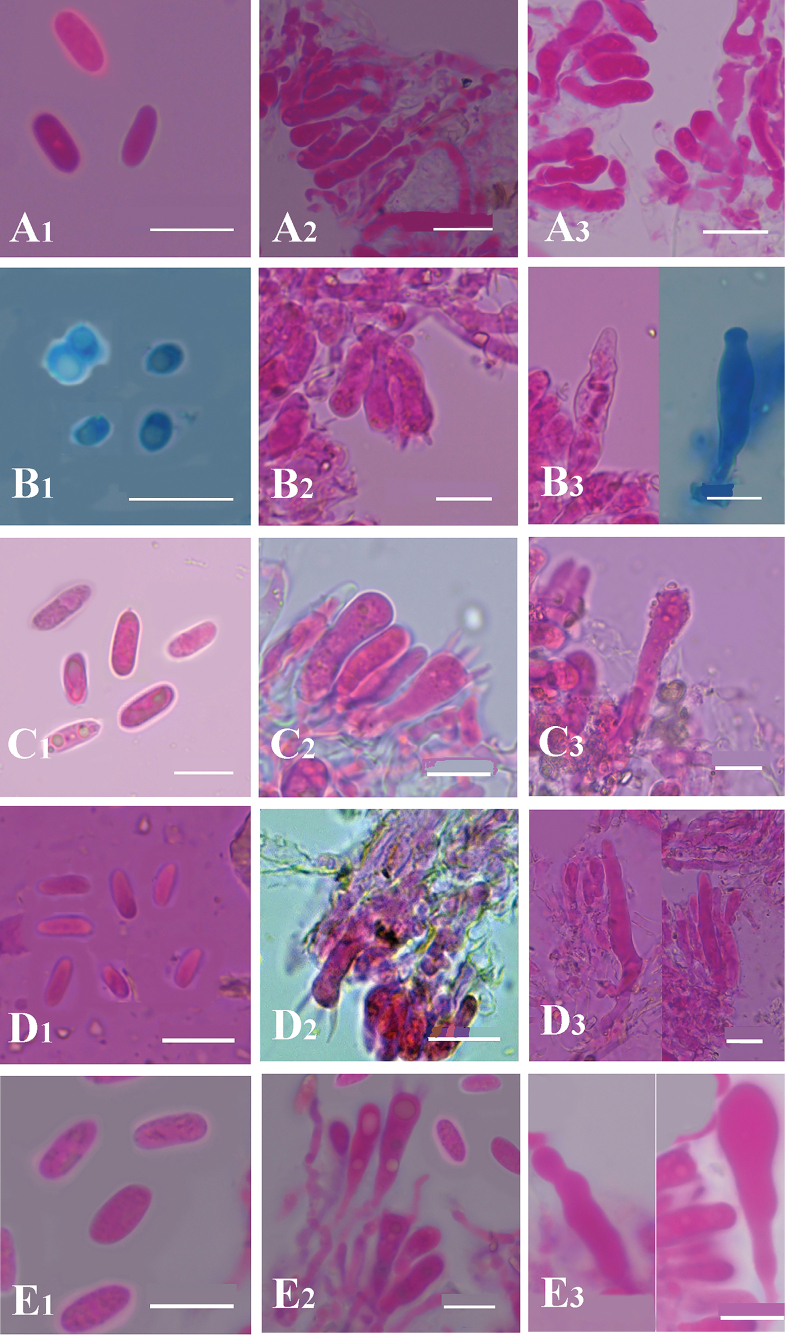
Comparison of micromorphological characteristics among the five new *Hyphoderma* species. **A1–A3**. *Hyphoderma alboarachnum*; **B1–B3**. *Hyphoderma bambusinum*; **C1–C3**. *Hyphoderma fulgens*; **D1–D3**. *Hyphoderma grandineum*; **E1–E3**. *Hyphoderma laceratum*. Scale bars: 10 μm (**A1–E3**).

Application of the pairwise homoplasy index (PHI) test to the combined partial ITS sequence dataset revealed no evidence of recombination among phylogenetically related species. No significant recombination events were observed between *H. alboarachnum* and *H. bambusinum* and *H. floccosum*, *H. setigerum*, *H. grandineum*, *H. transiens*, and other phylogenetically closely related species (Figs 2, 3). The test results for the combined partial ITS dataset showed that Φw = 1.0 (Φw > 0.05).

### Divergence time estimation for *Hyphoderma*

The results of divergence time estimation show (Figs 4, 5) that Polyporales emerged earlier, with a mean stem age of 163.71 Mya [95% highest posterior density (HPD) of 131.76–202.70 Mya], which is consistent with previous studies (Song and Cui 2017; Cui et al. 2025). Within Polyporales, *Hyphoderma* is closely related to the genera *Meripilus* and *Physisporinus*, with a mean stem age of 117.76 Mya (95% HPD of 92.38–147.74 Mya) and full support (1.0 PP; Figs 4, 5; Table 3).

**Table 3.** The estimated divergence times of the genus of *Hyphoderma*.

Taxa	Means of stem age (Ma)	95% HPD (Ma)
*Hyphoderma alboarachnum*	11.93	5.57–20.46
*H. amoenum*	32.59	16.57–49.20
*H. asianum*	81.14	53.20–106.54
*H. assimile*	40.40	24.36–57.71
*H. australosetigerum*	37.55	22.63–53.73
*H. bambusinum*	9.24	3.42–17.02
*H. cinereofuscum*	12.82	6.16–21.28
*H. cremeoalbum*	66.41	40.63–92.33
*H. crystallinum*	32.05	16.41–51.53
*H. definitum*	16.89	6.40–32.47
*H. fissuratum*	2.04	0.02–6.92
*H. floccosum*	21.55	11.21–33.35
*H. fulgens*	32.59	16.57–49.20
*H. granuliferum*	98.97	82.33–113.44
*H. grandineum*	27.49	15.22–41.59
*H. guangdongense*	17.61	7.59–29.35
*H. incrustatum*	47.30	23.50–74.28
*H. laceratum*	17.29	9.71–26.31
*H. litschaueri*	91.38	61.37–116.85
*H. macaronesicum*	9.49	3.33–18.47
*H. marginatum*	24.61	15.35–36.42
*H. medioburiense*	50.85	35.70–66.97
*H. membranaceum*	13.53	6.11–22.98
*H. microporoides*	79.38	54.13–104.43
*H. moniliforme*	18.85	8.07–32.66
*H. mopanshanense*	43.23	28.40–60.09
*H. nemorale*	112.25	96.42–121.98
*H. niveomarginatum*	30.86	18.16–45.31
*H. nudicephalum*	24.92	9.68–43.99
*H. obtusiforme*	66.27	52.57–84.85
*H. obtusum*	28.77	9.77–52.86
*H. occidentale*	16.89	6.40–32.47
*H. paramacaronesicum*	9.49	3.33–18.47
*H. pinicola*	17.61	7.59–29.35
*H. prosopidis*	40.03	19.71–65.11
*H. puerense*	18.85	8.07–32.66
*H. qujingense*	12.82	6.16–21.28
*H. roseocremeum*	2.04	0.02–6.92
*H. setigerum*	11.93	5.57–20.46
*H. sinense*	13.53	6.11–22.98
*H. sordidum*	24.92	9.68–43.99
*H. subsetigerum*	94.91	79.13–111.14
*H. tenuissimum*	32.93	17.60–48.15
*H. transiens*	9.24	3.42–17.02
*H. tropicum*	41.72	23.45–63.12
*H. variolosum*	22.31	9.77–38.21
*H. weishanense*	22.31	9.77–38.21
*H. yunnanense*	32.93	17.60–48.15

### The historical biogeography of *Hyphoderma*

The historical biogeography scenarios inferred using RASP are shown in Fig. 6. Results of the RASP analysis suggested that Asia is likely the center of origin for *Hyphoderma* species. Among these species, 39 are found in Asia, 18 in Europe, 14 in North America, four in South America, four in Africa, and six in Oceania, suggesting that Asia remains the center of *Hyphoderma* species. In addition, the ancestral state reconstruction dataset also suggested that Asia is the ancestral region for this genus (Fig. 6).

### Taxonomy

#### Hyphoderma alboarachnum

Taxon classificationFungiPolyporalesHyphodermataceae

W. Li and C.L. Zhao sp. nov.

928F33EF-A88C-555B-943E-BBB9832BCBA6

859913

Figs 8, 9

##### Typification.

China • Yunnan Province: Dehong, Yingjiang County, Tongbiguan Provincial Nature Reserve, GPS coordinates 24°30'N, 97°30'E, altitude 1006 m asl., on fallen angiosperm branch, leg. C.L. Zhao, 19 July 2023, CLZhao 30488 (SWFC 00030488), GenBank: ITS = PV470563.

**Figure 8.**
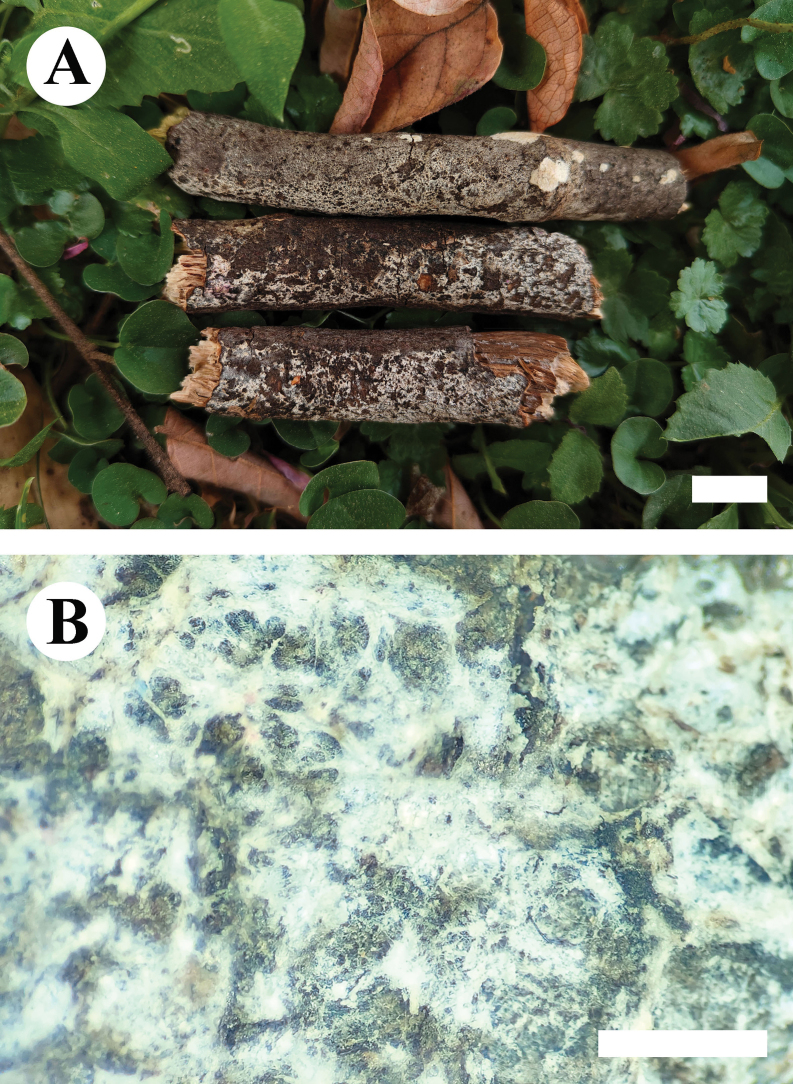
Basidiomata of *Hyphoderma alboarachnum* (holotype CLZhao 30488). Scale bars: 1 cm (**A**); 1 mm (**B**).

**Figure 9.**
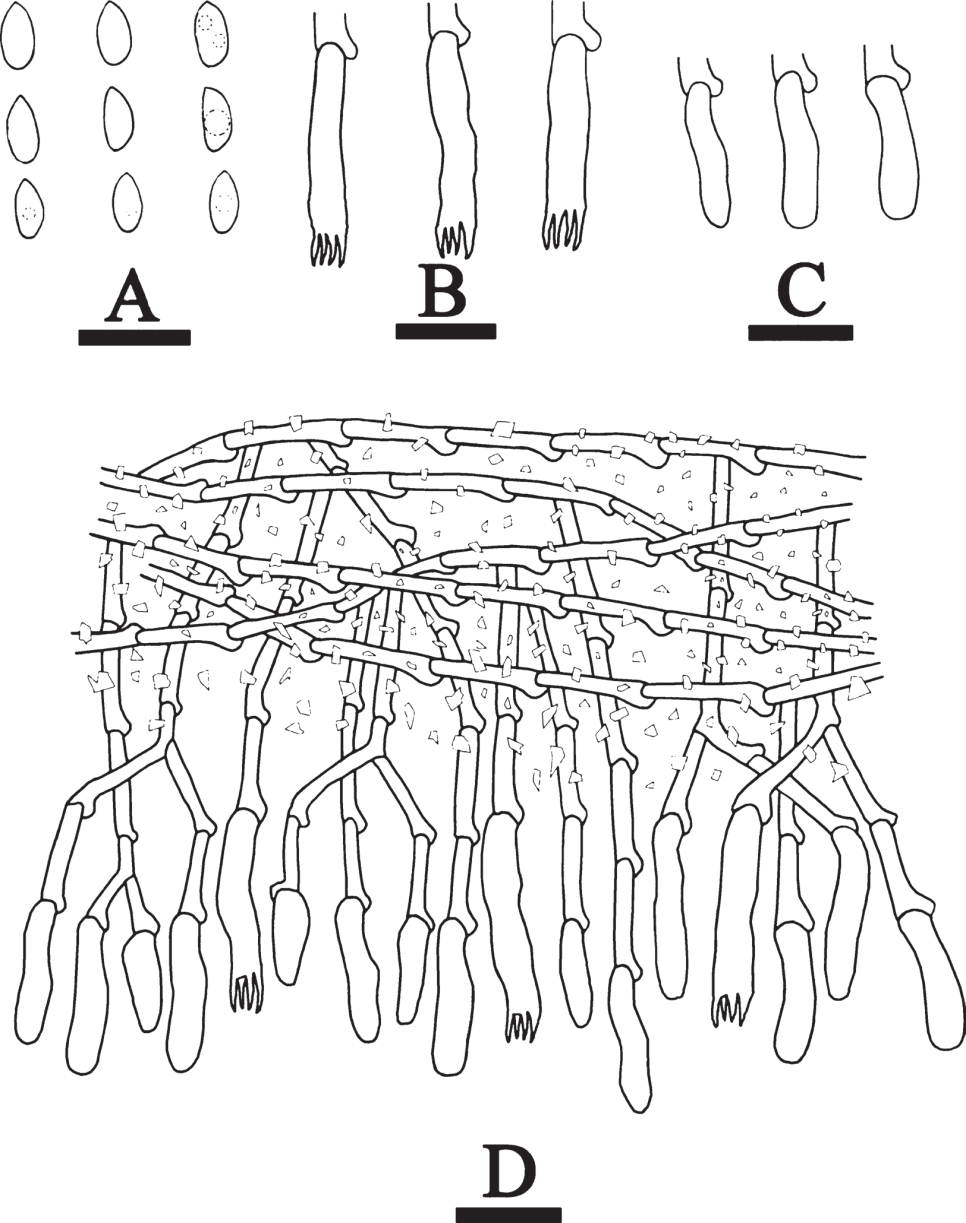
Microscopic structures of *Hyphoderma alboarachnum* (holotype CLZhao 30488). **A**. Basidiospores; **B**. Basidia; **C**. Basidioles; **D**. Part of a vertical section of the hymenium. Scale bars: 10 μm (**A–D**).

##### Etymology.

alboarachnum (Lat.): refers to the white basidiomata with an arachnoid hymenial surface of the type specimen.

##### Description.

***Basidiomata***. Annual, resupinate, adnate, membranaceous, without odor or taste when fresh, up to 12 cm long, 1.5 cm wide, and 100 μm thick. Hymenial surface arachnoid, white when fresh, becoming white to cream when dry. Sterile margin narrow, white, up to 1 mm.

***Hyphal system***. Monomitic; generative hyphae with clamp connections, colorless, thin-walled, branched, interwoven, 2.5–3.5 µm in diameter, IKI–, CB–, tissues unchanged in KOH.

***Hymenium***. Cystidia and cystidioles absent. Basidia cylindrical, colorless, thin-walled, smooth, 23.5–30 × 3.5–4.5 µm; basidioles numerous, similar to basidia in shape, but smaller.

***Spores***. Basidiospores oblong-ellipsoid to cylindrical, colorless, thin-walled, smooth, with inner oil droplets, IKI–, CB–, 5–6(–6.5) × 2–3 µm, L = 5.59 µm, W = 2.66 µm, Q = 2.25 (n = 30/1).

##### Notes.

In the phylogenetic analysis (Fig. 1), *Hyphoderma alboarachnum* (CLZhao 30488) was closely related to and formed a sister lineage with *H. floccosum* (CLZhao 17129, CLZhao 17215), with 100% ML, 91 MP bootstrap support, and a 1.00 BYPP value. However, morphologically, *H. floccosum* differs from *H. alboarachnum* by having a farinaceous hymenial surface and larger basidiospores (5–6 × 2–3 µm vs. 6–9.5 × 3–4.5 µm; Guan and Zhao 2021b). In addition, the latter was found in the Wenshan National Nature Reserve at an altitude of 2,480 m. Morphologically, *H. alboarachnum* is similar to *H. mopanshanense* and *H. membranaceum* in having subellipsoid to cylindrical basidiospores (Guan and Zhao 2021a; Ma et al. 2021). However, *H. mopanshanense* is distinguished from *H. alboarachnum* by its ceraceous hymenial surface and longer basidiospores (7.8–9.7 × 2.6–3.3 µm vs. 5–6 × 2–3 µm; Ma et al. 2021). The species *H. membranaceum* differs from *H. alboarachnum* by its tuberculate hymenial surface and larger basidiospores (11–13.5 × 4.5–5.5 µm vs. 5–6 × 2–3 µm; Guan and Zhao 2021a). Thus, based on morphological and phylogenetic evidence, we introduce our collection as a new species, *Hyphoderma alboarachnum*.

#### Hyphoderma bambusinum

Taxon classificationFungiPolyporalesHyphodermataceae

W. Li and C.L. Zhao sp. nov.

923F16F1-2C18-571F-8500-8187FA307E3D

859914

Figs 10, 11

##### Typification.

China • Yunnan Province: Dehong, Yingjiang County, Tongbiguan Provincial Nature Reserve, GPS coordinates 24°30'N, 97°30'E, altitude 1006 m asl., on dead bamboo, leg. C.L. Zhao, 18 July 2023, CLZhao 29903 (SWFC 00029903), GenBank: ITS = PV469674; nLSU = PV819428.

**Figure 10.**
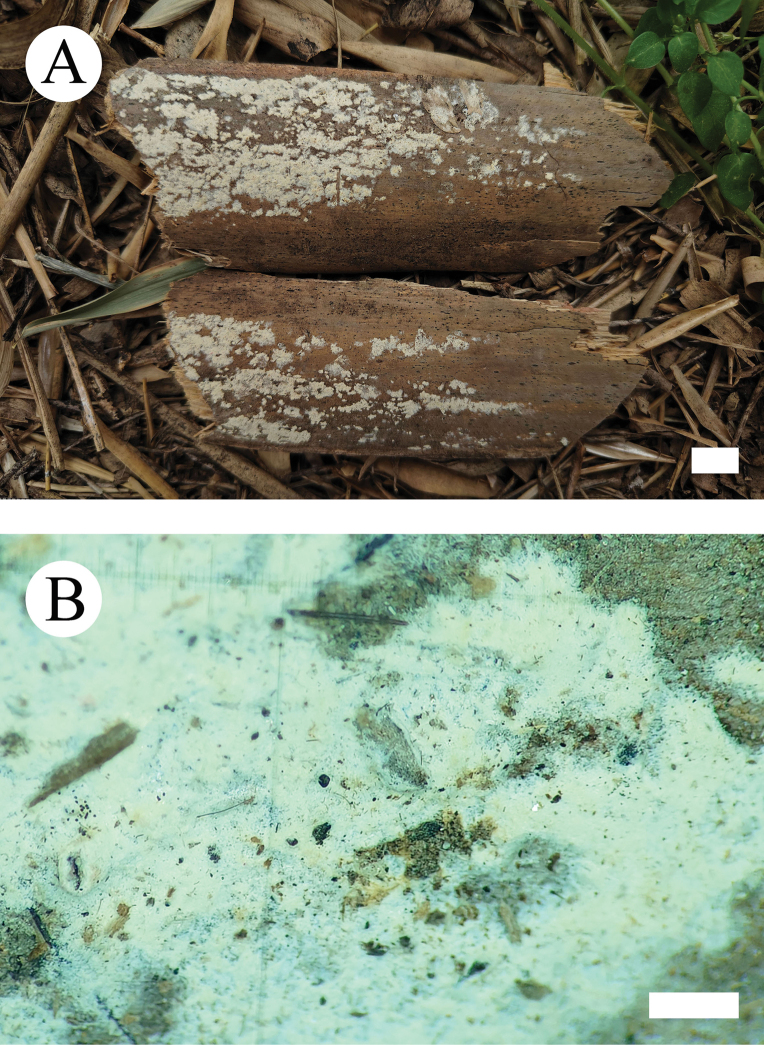
Basidiomata of *Hyphoderma bambusinum* (holotype CLZhao 29903). Scale bars: 1 cm (**A**); 1 mm (**B**).

**Figure 11.**
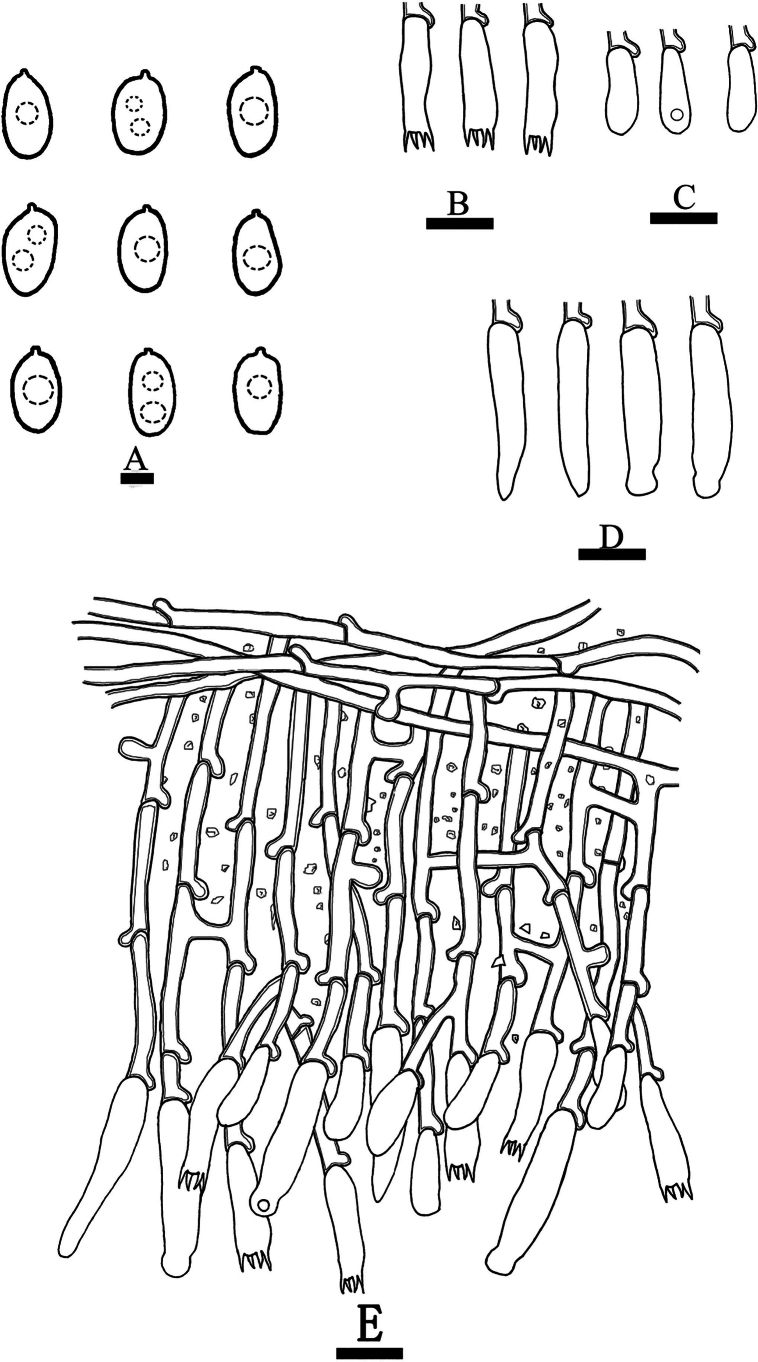
Microscopic structures of *Hyphoderma bambusinum* (holotype CLZhao 29903). **A**. Basidiospores; **B**. Basidia; **C**. Basidioles; **D**. Cystidia; **E**. Part of a vertical section of the hymenium. Scale bars: 2 μm (**A**); 10 μm (**B–E**).

##### Etymology.

bambusinum (Lat.) refers to the host bamboo on which the fungal species grows.

##### Description.

***Basidiomata***. Annual, resupinate, adnate, membranaceous, without odor or taste when fresh, up to 7 cm long, 2.5 cm wide, and 100 μm thick. Hymenial surface smooth, white to cream when fresh, becoming cream to buff when dry. Sterile margin narrow, white to cream, up to 1 mm.

***Hyphal system***. Monomitic; generative hyphae with clamp connections, colorless, thick, branched, interwoven, 2.5–3 µm in diameter, IKI–, CB–, tissues unchanged in KOH.

***Hymenium***. Cystidia tubular, colorless, thin-walled, smooth, 21.5–26.5 × 5–6.5 µm. Basidia subclavate to subcylindrical, thin-walled, smooth, slightly flexuous, with four sterigmata and a simple septum at the base, 16–17.5 × 3.5–5.5 µm; basidioles similar to basidia in shape but slightly smaller.

***Spores***. Basidiospores ellipsoid, colorless, thin-walled, smooth, with oil droplets inside, IKI–, CB–, 4.5–6.5 × 3–4.5(–5) µm, L = 5.55 µm, W = 3.78 µm, Q = 1.47 (n = 30/1).

##### Notes.

*Hyphoderma bambusinum* (CLZhao 29903) was found to be phylogenetically closely related to *H. transiens* (NH 12304 [GB]), with 100% ML, 100 MP bootstrap support, and a 1.00 BYPP value. However, morphologically, *H. transiens* differs from *H. bambusinum* by having an odontioid hymenial surface and longer basidiospores (9–13 × 3–4.5 µm vs. 4.5–6.5 × 3–4.5 µm; Parmasto 1968); additionally, the latter was found in Portugal and growing on the bark of *Quercus*. Morphologically, *H. bambusinum* is similar to *H. cremeoalbum* (Höhn. and Litsch.) Jülich. and *H. floccosum* by having ellipsoid basidiospores (Bernicchia and Gorjón 2010; Guan and Zhao 2021b). However, *H. cremeoalbum* is distinguished from *H. bambusinum* by its larger basidiospores (10–14 × 5–6.5 µm vs. 4.5–6.5 × 3–4.5 µm; Bernicchia and Gorjón 2010). The species *H. floccosum* differs from *H. bambusinum* by its ceraceous basidiomata and longer, tubular cystidia (37.5–100 × 4–8.5 µm vs. 21.5–26.5 × 5–6.5 µm; Guan and Zhao 2021b). These morphological differences, together with phylogenetic analyses, support the conclusion that our taxon represents a new species, *Hyphoderma bambusinum*.

#### Hyphoderma fulgens

Taxon classificationFungiPolyporalesHyphodermataceae

W. Li and C.L. Zhao sp. nov.

C69F8956-7D84-5290-B5AE-3266E1367A48

859915

Figs 12, 13

##### Typification.

China • Yunnan Province: Dehong, Yingjiang County, Tongbiguan Provincial Nature Reserve, GPS coordinates 24°30'N, 97°30'E, altitude 1006 m asl., on fallen angiosperm branch, leg. C.L. Zhao, 3 July 2024, CLZhao 37429 (SWFC 00037429), GenBank: ITS = PV829544; nLSU = PV810095.

**Figure 12.**
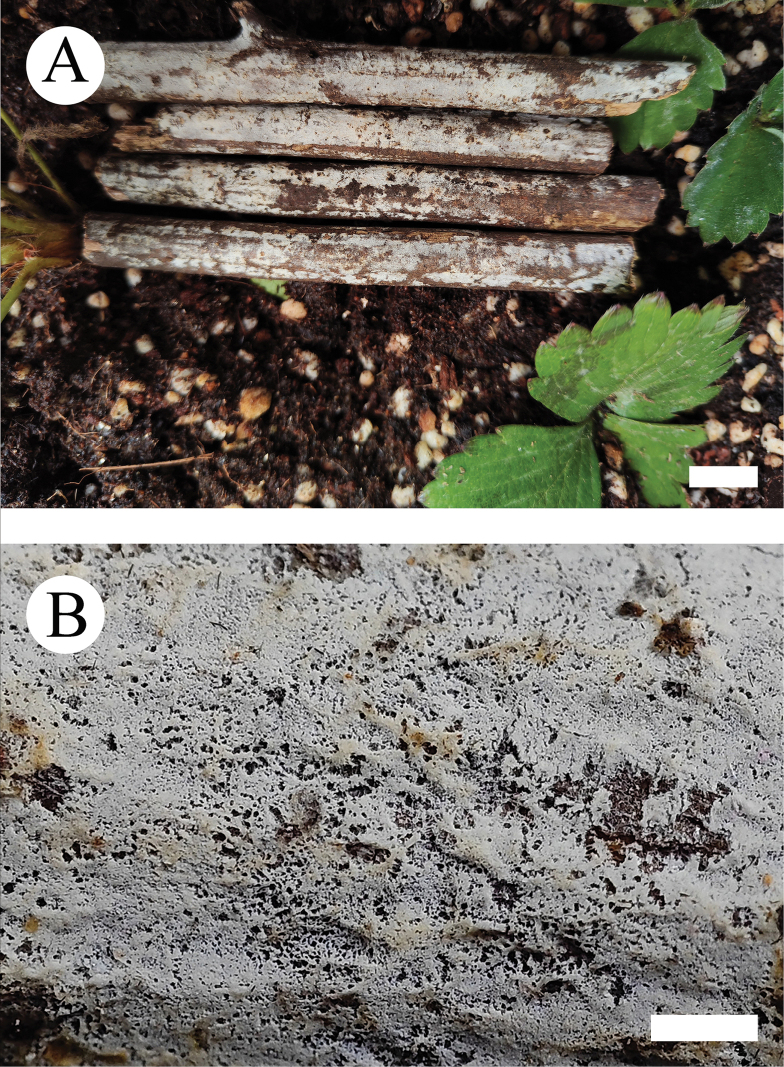
Basidiomata of *Hyphoderma fulgens* (holotype CLZhao 37429). Scale bars: 1 cm (**A**); 1 mm (**B**).

**Figure 13.**
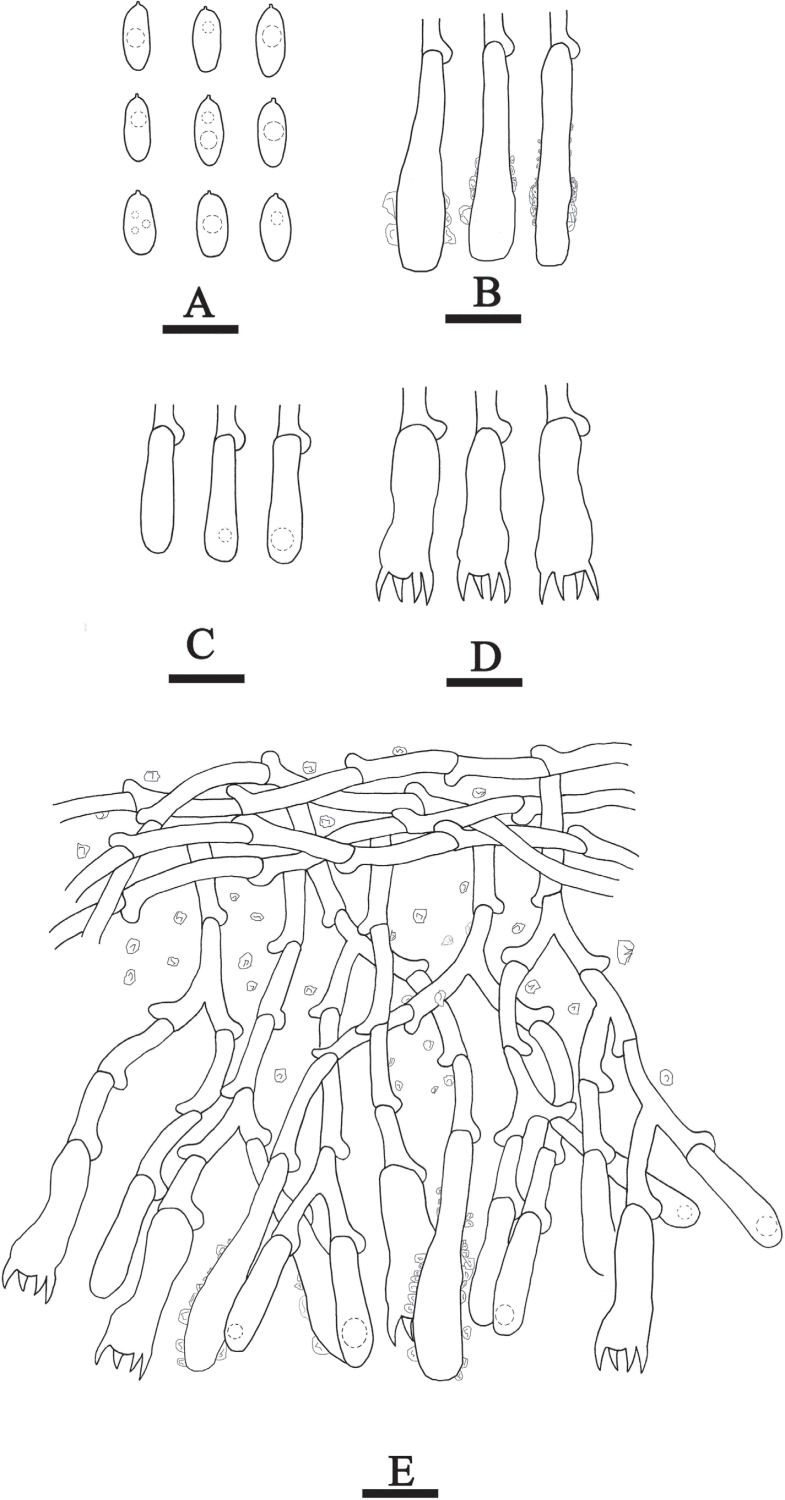
Microscopic structures of *Hyphoderma fulgens* (holotype CLZhao 37429). **A**. Basidiospores; **B**. Cystidia; **C**. Basidioles; **D**. Basidia; **E**. Part of a vertical section of the hymenium. Scale bars: 10 μm (**A–E**).

##### Etymology.

fulgens (Lat.): refers to the shiny color of the hymenial surface of the specimens.

##### Description.

***Basidiomata***. Annual, resupinate, adnate, membranaceous, without odor or taste when fresh, up to 9 cm long, 1.8 cm wide, and 100 μm thick. Hymenial surface smooth, white to cream when fresh, white when dry. Sterile margin narrow, white, up to 1 mm.

***Hyphal system***. Monomitic; generative hyphae with clamp connections, colorless, thin-walled, branched, interwoven, 2.5–3.2 µm in diameter, IKI–, CB–, tissues unchanged in KOH.

***Hymenium***. Cystidia clavate, colorless, thin-walled, smooth, encrusted, 30.4–37.1 × 5.5–7.5 µm. Basidia barreled, thin-walled, smooth, slightly flexuous, with four sterigmata and a simple septum at the base, 21.5–26 × 5.5–8 µm; basidioles similar to basidia in shape but slightly smaller.

***Spores***. Basidiospores subcylindrical, colorless, thin-walled, smooth, with oil droplets inside, IKI–, CB–, (7–)7.5–11.5 × (2–)2.5–4 µm, L = 9.55 µm, W = 3.39 µm, Q = 2.74–2.89 (n = 120/4).

##### Additional specimens examined (paratypes).

China • Yunnan Province: Dehong, Yingjiang County, Tongbiguan Provincial Nature Reserve, GPS coordinates 24°30'N, 97°30'E, altitude 1006 m asl., on fallen angiosperm branch, leg. C.L. Zhao, 19 July 2023, CLZhao 30254 (SWFC 00030254); 2 July 2024, CLZhao 37266 (SWFC 00037266); • Mang City, Tongbiguan Provincial Nature Reserve, GPS coordinates 24°42'N, 97°54'E, altitude 1006 m asl., on fallen angiosperm branch, leg. C.L. Zhao, 29 June 2024, CLZhao 36600 (SWFC 00036600); 8 July 2024, CLZhao 39677 (SWFC 00039677); CLZhao 39618 (SWFC 00039618); CLZhao 39474 (SWFC 00039474).

##### Notes.

In the phylogenetic analysis, the specimens of *Hyphoderma fulgens* (CLZhao 37429, CLZhao 30254, CLZhao 36600, CLZhao 39474) formed a closely related sister relationship to *H. amoenum* (Burt) Donk (USO 286622), with 99% ML, 89 MP bootstrap support, and a 0.99 BYPP value. However, morphologically, *H. amoenum* differs from *H. fulgens* by having wider basidiospores (9–13 × 5–6 µm vs. 7.5–11.5 × 2.5–4 µm; Tellería et al. 2012). Morphologically, *H. fulgens* is similar to *H. guangdongense* and *H. tenuissimum* by having subcylindrical basidiospores (Guan and Zhao 2021a; Su et al. 2024). However, *H. guangdongense* is distinguished from *H. fulgens* by its farinaceous hymenial surface and septate cystidia (Su et al. 2024). In addition, *H. tenuissimum* differs from *H. fulgens* by its tuberculate to minutely grandinioid hymenial surface and larger cystidia (50–220 × 6.5–13 µm vs. 30.4–37.1 × 5.5–7.5 µm; Guan and Zhao 2021a). These morphological and phylogenetic data indicate that our collections represent a new species, *Hyphoderma fulgens*.

#### Hyphoderma grandineum

Taxon classificationFungiPolyporalesHyphodermataceae

W. Li and C.L. Zhao sp. nov.

CBC6C098-6CC3-59C5-BD14-178642221919

859916

Figs 14, 15

##### Typification.

China • Yunnan Province: Dehong, Yingjiang County, Tongbiguan Provincial Nature Reserve, GPS coordinates 24°30'N, 97°30'E, altitude 1006 m asl., on the fallen angiosperm branch, leg. C.L. Zhao, 18 July 2023, CLZhao 30046 (SWFC 00030046), GenBank: ITS = PV470561, nLSU = PV819429.

**Figure 14.**
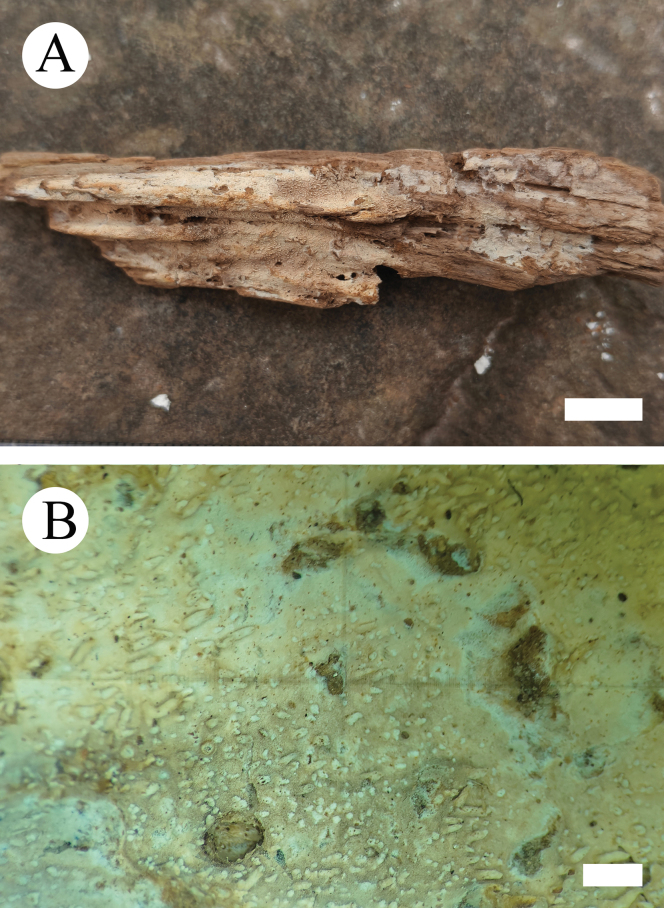
Basidiomata of *Hyphoderma grandineum* (holotype CLZhao 30046). Scale bars: 1 cm (**A**); 1 mm (**B**).

**Figure 15.**
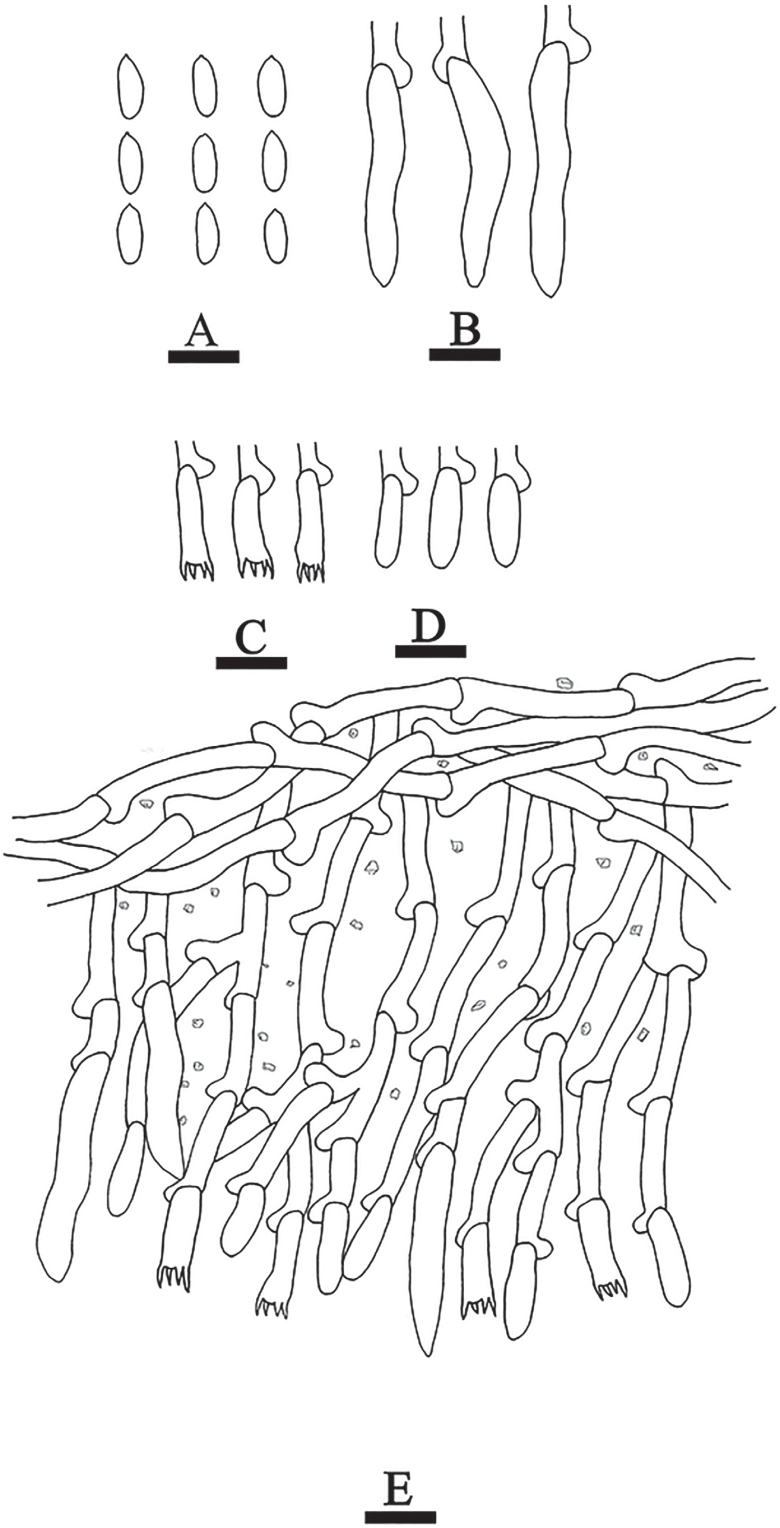
Microscopic structures of *Hyphoderma grandineum* (holotype CLZhao 30046). **A**. Basidiospores; **B**. Cystidia; **C**. Basidia; **D**. Basidioles; **E**. Part of a vertical section of the hymenium. Scale bars: 10 μm (**A–E**).

##### Etymology.

grandineum (Lat.): refers to the grandinioid hymenial surface of the type specimen.

##### Description.

***Basidiomata***. Annual, resupinate, adnate, membranaceous, without odor or taste when fresh, up to 7.5 cm long, 2 cm wide, and 100 μm thick. Hymenial surface grandinioid, cream when fresh, becoming cream to yellowish when dry. Sterile margin narrow, white, up to 1 mm.

***Hyphal system***. Monomitic; generative hyphae with clamp connections, colorless, thin-walled, branched, interwoven, 3–3.6 µm in diameter, IKI–, CB–, tissues unchanged in KOH.

***Hymenium***. Leptocystidia, colorless, thin-walled, smooth, 29.5–33.5 × 4–5 µm. Basidia clavate, thin-walled, smooth, slightly flexuous, with four sterigmata and a simple septum at the base, 18–19.5 × 4.5–6 µm; basidioles similar to basidia in shape but slightly smaller.

***Spores***. Basidiospores cylindrical, colorless, thin-walled, smooth, IKI–, CB–, (6–)7–9 × 2.5–4 µm, L = 7.9 µm, W = 3.2 µm, Q = 2.48–2.5 (n = 60/2).

##### Additional specimen examined (paratype).

China • Yunnan Province: Dehong, Ruili City, Tongbiguan Provincial Nature Reserve, GPS coordinates 23°22'N, 97°18'E, altitude 976 m asl., on the fallen angiosperm branch, leg. C.L. Zhao, 2 December 2024, CLZhao 43328(SWFC 00043328), GenBank: ITS = PV470562, nLSU = PV819430.

##### Notes.

In the phylogenetic analysis, *Hyphoderma grandineum* (CLZhao 30046, CLZhao 43328) formed a closely related sister relationship with the clade consisting of *H. transiens* (Bres.) Parmasto (NH 12304 [GB]) and *H. bambusinum* (CLZhao 29903), with 100% ML, 100 MP bootstrap support, and a 1.00 BYPP value. However, morphologically, *H. transiens* differs from *H. grandineum* by having a whitish to ochraceous hymenial surface and longer basidiospores (9–13 × 3–4.5 µm vs. 7–9 × 2.5–4 µm; Parmasto 1968); in addition, the latter was found in Portugal. Morphologically, *H. grandineum* is similar to *H. tropicum* and *H. tenuissimum* by having a tuberculate to odontoid hymenial surface (Guan et al. 2021; Duan et al. 2023). However, *H. tropicum* is distinguished from *H. grandineum* by its coriaceous hymenial surface and moniliform cystidia (Duan et al. 2023). In addition, *H. tenuissimum* differs from *H. grandineum* by its larger cystidia (50–220 × 6.5–13 µm vs. 29.5–33.5 × 4–5 µm; Guan et al. 2021). These morphological and phylogenetic evidences show that our collections represent a new species, *Hyphoderma grandineum*.

#### Hyphoderma laceratum

Taxon classificationFungiPolyporalesHyphodermataceae

W. Li and C.L. Zhao sp. nov.

244B55E2-697E-5829-87E3-95218FA73B9B

861118

Figs 16, 17

##### Typification.

China • Yunnan Province: Diqing, Weixi County, Weideng Town, Fuchuan Village, GPS coordinates 27°17'N, 99°16'E, altitude 1700 m asl., on the fallen angiosperm branch, leg. C.L. Zhao, 12 October 2023, CLZhao 34242 (SWFC 00034242), GenBank: ITS = PV829552, nLSU = PV810101.

**Figure 16.**
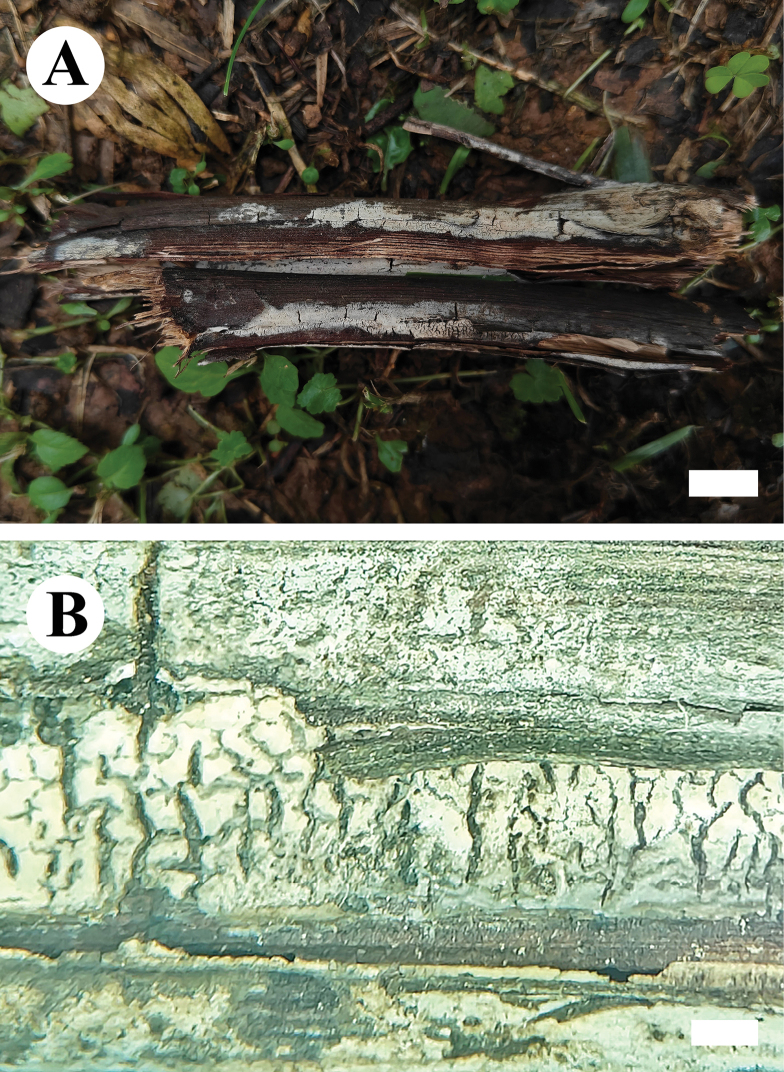
Basidiomata of *Hyphoderma laceratum* (holotype CLZhao 34242). Scale bars: 1 cm (**A**); 1 mm (**B**).

**Figure 17.**
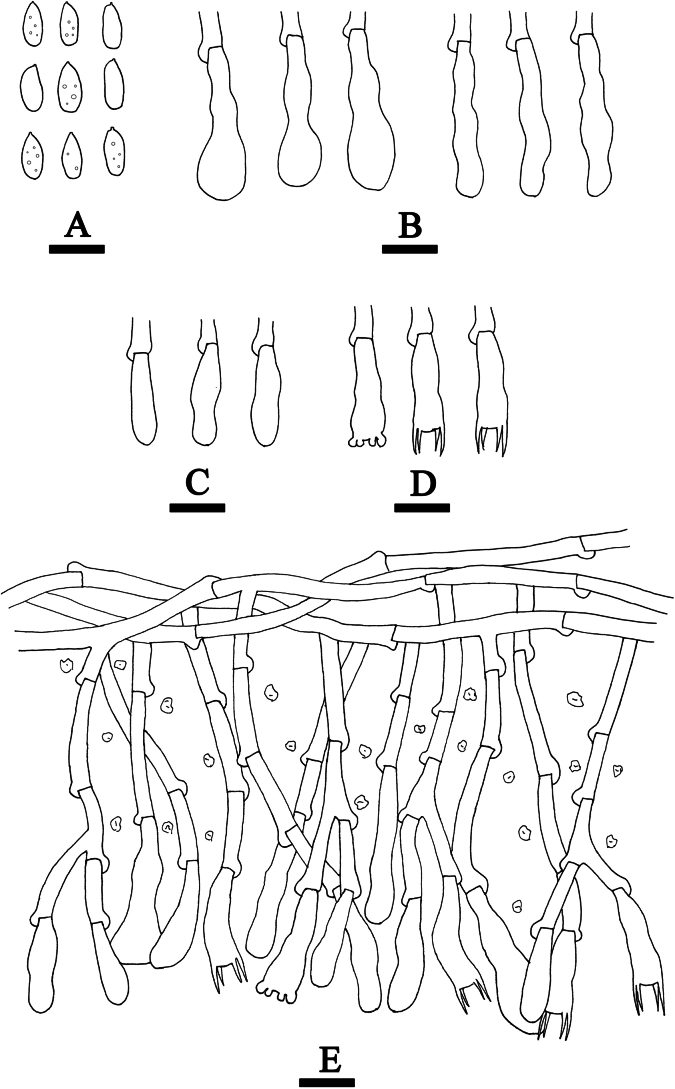
Microscopic structures of *Hyphoderma laceratum* (holotype CLZhao 34242). **A**. Basidiospores; **B**. Cystidia; **C**. Basidioles; **D**. Basidia; **E**. Part of a vertical section of the hymenium. Scale bars: 10 μm (**A–E**).

##### Etymology.

laceratum (Lat.): refers to the lacerate hymenial surface of the type specimens.

##### Description.

***Basidiomata***. Annual, resupinate, adnate, membranaceous, without odor or taste when fresh, up to 10.5 cm long, 2.5 cm wide, and 150 μm thick. Hymenial surface smooth, white to cream when fresh, becoming cream when dry. Sterile margin narrow, white, up to 1 mm.

***Hyphal system***. Monomitic; generative hyphae with clamp connections, colorless, thin-walled, branched, interwoven, 2.5–3.3 µm in diameter, IKI–, CB–, tissues unchanged in KOH.

***Hymenium***. Cystidia, two types: (1) Clavate, colorless, thin-walled, smooth, 27–29 × 6.5–8 µm; (2) Capitate, colorless, thin-walled, smooth, 26–30 × 7.5–8.5 µm. Basidia subcylindrical, thin-walled, smooth, slightly flexuous, with four sterigmata and a simple septum at the base, 18–20.5 × 5–7 µm; basidioles similar to basidia in shape but slightly smaller.

***Spores***. Basidiospores sub-ellipsoid, colorless, thin-walled, smooth, with guttulate, IKI–, CB–, (6–)6.5–9 × (2.5–)3–4(–4.5) µm, L = 7.72 µm, W = 3.49 µm, Q = 2.18–2.35 (n = 330/11).

##### Additional specimens examined (paratypes).

China • Yunnan Province: Diqing, Weixi County, Weideng Town, Fuchuan Village, GPS coordinates 27°17'N, 99°16'E, altitude 1700 m asl., on the fallen angiosperm branch, leg. C.L. Zhao, 12 October 2023, CLZhao 34163 (SWFC 00034163), CLZhao 34138 (SWFC 00034138); • Zhonglu Town, GPS coordinates 27°17'N, 99°16'E, altitude 1811 m asl., on the fallen angiosperm branch, leg. C.L. Zhao, 15 October 2023, CLZhao 34961 (SWFC 00034961), CLZhao 35040 (SWFC 00035040), CLZhao 34718 (SWFC 00034718), CLZhao 34672 (SWFC 00034672), CLZhao 34958 (SWFC 00034958); • Qujing, Zhanyi District, Lingjiao Town, Xiajia Village, GPS coordinates 25°8'N, 103°6'E, altitude 2040 m asl., on the fallen angiosperm branch, leg. C.L. Zhao, 6 March 2023, CLZhao 27226 (SWFC 00027226); • Dehong, Yingjiang County, Tongbiguan Provincial Nature Reserve, GPS coordinates 24°30'N, 97°30'E, altitude 1006 m asl., on the fallen angiosperm branch, leg. C.L. Zhao, 19 July 2023, CLZhao 30414 (SWFC 00030414), CLZhao 30552 (SWFC 00030552).

##### Notes.

In the phylogenetic analysis, *Hyphoderma laceratum* (CLZhao 34958, CLZhao 34672, CLZhao 34961, CLZhao 34242) formed a closely related sister relationship with *H. marginatum* (CLZhao 3404), with 99% ML bootstrap support and a 0.99 BYPP value. However, morphologically, *H. marginatum* differs from *H. laceratum* by having a cream hymenial surface and longer basidia (21–31.5 × 5–7 µm vs. 18–20.5 × 5–7 µm; Duan et al. 2023). Morphologically, *H. laceratum* is similar to *H. niveomarginatum* by having a cream hymenial surface (Yang et al. 2023). Further, *H. niveomarginatum* is distinguished from *H. laceratum* by its moniliform cystidia (Yang et al. 2023). Based on these morphological differences and phylogenetic analysis, we propose it as a new species, *Hyphoderma laceratum*.

## Discussion and conclusion

Many recently described wood-decaying fungal taxa have been reported from subtropical and tropical regions, and the genus *Hyphoderma* represents one of the most rapidly expanding groups in these areas (Nilsson et al. 2003; Wu et al. 2010; Martín et al. 2018; Guan and Zhao 2021a, b). Molecular systematics and taxonomy are essential for resolving diversity within *Hyphoderma* because species in this genus exhibit substantial variation in basidiomata morphology, including farinaceous, coriaceous, and membranaceous forms, as well as hymenophore types ranging from smooth to grandinioid (Guan et al. 2023; Su et al. 2024; Li et al. 2025; Yang et al. 2025b). Despite this variation, the hymenial surface predominantly ranges from cream to whitish (Yurchenko and Wu 2014a, b; Guan et al. 2023; Wang et al. 2025). The morphological delineation of *Hyphoderma* species is challenging, and continuous descriptions of new taxa further reduce the diagnostic value of the limited morphological characteristics available for distinguishing species within each morpho-ecological group (Yang et al. 2025b). In the present study, we described five new species, viz., *H. alboarachnum*, *H. bambusinum*, *H. fulgens*, *H. grandineum*, and *H. laceratum*. These taxa represent distinct hymenophore types and highlight the necessity of integrative taxonomy for accurate species identification.

Wood-decaying fungi occupy a broad range of substrates, including living trees, decorticated wood of dead branches and trunks, and processed wood materials (Dai et al. 2015; Cui et al. 2019; Zhao et al. 2023; Zhang et al. 2023; He et al. 2024; Hyde et al. 2024; Luo et al. 2024; Deng et al. 2025; Yang et al. 2025a). Recent biogeographical studies, particularly in Polyporaceae and Hymenochaetaceae, have substantially improved our understanding of diversification patterns among wood-decaying fungi (Yang et al. 2023; Zhao et al. 2025). Consistent with these findings, our results show that the geographic distribution of *Hyphoderma* follows a distinct and well-structured biogeographical pattern, similar to that observed in other major groups of wood-decaying fungi (Song and Cui 2017; Yang et al. 2023).

Molecular dating analyses have provided important insights into the evolution of wood-decaying fungi (Zhao et al. 2025). According to our divergence-time estimates based on ITS and nLSU sequence datasets, *Hyphoderma* likely originated during the Cretaceous period. The mean stem age is 117.76 Mya (95% HPD = 92.38–147.74 Mya), with strong support (PP = 1.0). This deep evolutionary origin is consistent with the long-term ecological continuity of Cretaceous forest ecosystems (Martín et al. 2018; Cui et al. 2025). Our biogeographical reconstruction further suggests that *Hyphoderma* most likely originated in Asia, a recognized global biodiversity hotspot (Cui et al. 2025). Dispersal between East Asia and North America may have occurred through Beringia (Cui et al. 2025; Zhao et al. 2025). In contrast, later vicariance events, such as the opening of the Bering Strait, probably restricted gene flow between the Old and New Worlds and contributed to the present distribution of the genus (Hibbett 2001; Cai et al. 2014; Zhao et al. 2025).

In summary, by combining extensive field collections, detailed morphological observations, and multi-locus phylogenetic analyses, we reconstructed the evolutionary relationships within *Hyphoderma* and uncovered five previously unknown species. Our divergence-time and biogeographical analyses provide a refined understanding of the evolutionary origin and historical distribution of the genus, thereby contributing valuable insights into the broader evolutionary history of wood-decaying fungi. The present study fills knowledge gaps regarding wood-inhabiting fungi by reporting new taxa and providing detailed morphological descriptions and phylogenetic analyses while contributing to the enrichment of fungal diversity in Asia.

## Supplementary Material

XML Treatment for Hyphoderma alboarachnum

XML Treatment for Hyphoderma bambusinum

XML Treatment for Hyphoderma fulgens

XML Treatment for Hyphoderma grandineum

XML Treatment for Hyphoderma laceratum

## Additional information

### Use of AI

No use of AI was reported.

### Author ORCIDs

Wen Li https://orcid.org/0009-0005-1394-4945

Haijiao Wang https://orcid.org/0009-0008-7690-2576

Xiyan Wang https://orcid.org/0009-0006-9978-1901

Xiuhe Liao https://orcid.org/0009-0005-8288-0340

Kaisheng Wang https://orcid.org/0009-0007-4840-816X

Wijesinghe Arachchige Subodini Nuwanthika https://orcid.org/0000-0002-5625-9578

Changlin Zhao https://orcid.org/0000-0002-8668-1075

### Data availability

All of the data that support the findings of this study are available in the main text.
